# Multiple bacterial virulence factors focused on adherence and biofilm formation associate with outcomes in cirrhosis

**DOI:** 10.1080/19490976.2021.1993584

**Published:** 2021-11-08

**Authors:** Jasmohan S Bajaj, Amirhossein Shamsaddini, Chathur Acharya, Andrew Fagan, Masoumeh Sikaroodi, Edith Gavis, Sara McGeorge, Alexander Khoruts, Michael Fuchs, Richard K Sterling, Hannah Lee, Patrick M Gillevet

**Affiliations:** a Gastroenterology, Hepatology and Nutrition, Virginia Commonwealth University and Central Virginia Veterans Healthcare System, Richmond, Virginia, USA; b Microbiome Analysis Center, George Mason University, Manassas, Virginia, USA; c Gastroenterology, Hepatology and Nutrition, Center for Immunology and Biotechnology Institute, University of Minnesota, Minneapolis, Minnesota, USA

**Keywords:** Hospitalizations, death, infections, enterococcus, fecal microbiota transplant

## Abstract

**Background & Aims:**

Altered gut microbiota is associated with poor outcomes in cirrhosis, including infections and hepatic encephalopathy (HE). However, the role of bacterial virulence factors (VFs) is unclear. Aim: Define association of VFs with cirrhosis severity and infections, their linkage with outcomes, and impact of fecal microbiota transplant (FMT).

**Methods:**

VF abundances were determined using metagenomic analysis in stools from controls and cirrhosis patients (compensated, HE-only, ascites-only, both and infected). Patients were followed for 90-day hospitalizations and 1-year death. Stool samples collected before/after a placebo-controlled FMT trial were also analyzed. Bacterial species and VFs for all species and selected pathogens (*Escherichia, Klebsiella, Pseudomonas, Staphylococcus, Streptococcus*, and *Enterococcus* spp) were compared between groups. Multi-variable analyses were performed for clinical biomarkers and VFs for outcome prediction. Changes in VFs pre/post-FMT and post-FMT/placebo were analyzed. *Results*: We included 233 subjects (40 controls, 43 compensated, 30 HE-only, 20 ascites-only, 70 both, and 30 infected). Decompensated patients, especially those with infections, had higher VFs coding for siderophores, biofilms, and adhesion factors versus the rest. Biofilm and adhesion VFs from *Enterobacteriaceae* and *Enterococcus* spp associated with death and hospitalizations independent of clinical factors regardless of when all VFs or selected pathogens were analyzed. FMT was associated with reduced VF post-FMT versus pre-FMT and post-placebo groups.

**Conclusions:**

Virulence factors from multiple species focused on adhesion and biofilms increased with decompensation and infections, associated with death and hospitalizations independent of clinical factors, and were attenuated with FMT. Strategies focused on targeting multiple virulence factors could potentially impact outcomes in cirrhosis.

**Presentations:**

Portions of this manuscript were an oral presentation in the virtual International Liver Congress 2021

**Abbreviations:**

VF: virulence factors, HE: hepatic encephalopathy, FMT: Fecal microbiota transplant, PPI: proton pump inhibitors, LPS: lipopolysaccharides, VFDB: Virulence factor database, OTU: operational taxonomic units, SBP: spontaneous bacterial peritonitis, UTI: urinary tract infections, MRSA: methicillin resistant Staphylococcus aureus, VRE: vancomycin-resistant Enterococcus, MAAsLin2: Microbiome Multivariable Associations with Linear Models, LPS: lipopolysaccharides, AKI: acute kidney injury

## Introduction:

Patients with cirrhosis have a high risk of progression to complications, such as hepatic encephalopathy (HE), ascites, and infections. This progression is associated with gut microbial changes in the bacteria, fungi, and bacteriophages.^1^^, 2^ With worsening disease, there is a higher relative abundance of potential pathogenic taxa belonging to Enterobacteriaceae and Enterococcaceae families and lower autochthonous taxa such as Lachnospiraceae and Ruminococcaceae.^3^ Specifically, pathogenic *Escherichia, Klebsiella, Pseudomonas, Enterococcus, Streptococcus*, and *Staphylococcus* spp cause most infections in patients with cirrhosis.^4,5^ Current therapies directed at the microbiota in cirrhosis include antibiotics, pre-biotics, and fecal microbiota transplants (FMT), all of which are relatively broad tools.^6^ Focusing on specific taxa and factors that contribute to disease pathogenesis is needed to make therapies focused on the microbiota precise and individualized.

A major target for potential focused therapies could be the carriage and expression of virulence factor genes.^7–9^ These virulence factors (VFs) are defined as gene products that enable microorganisms to colonize, proliferate, and cause damage to the host.^10^ Conventional VFs, which can directly contribute to the disease process, include secreted proteins, such as protein toxins and enzymes, and cell-surface structures, such as capsular polysaccharides, lipopolysaccharides (LPS), and outer membrane proteins, which directly contribute to the disease processes.^9,11,12^ However, several other genes encoding virulence traits, such as secretion machinery, siderophores, catalases, regulators, etc., are indirectly involved in pathogenesis, may also be relevant for bacteria to establish infection or precipitate poor outcomes.^10^

Understanding the range and burden of specific VFs is necessary for the development of targeted therapies that can provide maximal benefit with lesser risk in patients with cirrhosis. We aimed to determine the carriage of VFs in a cohort of subjects ranging from healthy controls through compensated, decompensated outpatients and inpatients with infections. We then performed multivariable analyses to determine the independent contribution of VFs to clinically relevant outcomes of death and hospitalizations. Finally, changes in VFs using oral FMT determined in the prior trial were evaluated.

## Results:

### Non-Trial Cohort:

#### Subject information:

We included 40 healthy controls and 193 patients with cirrhosis. Of these, 43 were compensated, 30 had HE only, 20 only ascites, 70 had both ascites and HE, and 30 were inpatients with SBP or UTI. As shown in table 1, groups were different with respect to cirrhosis severity and medication use. However, demographics and diabetes rates were similar. Infected and patients with both HE and ascites had higher hospitalizations and death compared to the rest.

**Table 1.** Characteristics of Subject Groups in the Non-Trial Cohort

	Controls (n = 40)	All Cirrhosis (n = 193)					P-value all groups
		Compensated (n = 43)	Decompensated Cirrhosis (n = 120)			Infected (N = 30)	
			HE only (n = 30)	Ascites only (n = 20)	Both (n = 70)		
Age	58.6 ± 10.3	60.3 ± 7.2	61.0 ± 11.4	59.2 ± 10.1	60.1 ± 8.9	55.6 ± 11.4	0.28
Caucasian/ African American/ Other	26/13/1	31/12/0	23/7/0	15/5/0	49/18/3	22/7/0	0.89
Latinx/Not	5/35	2/41	3/27	2/18	7/63	2/28	0.86
Gender (male)	24 (60%)	36 (84%)	15 (50%)	16 (80%)	59 (84%)	22 (73%)	0.30
Proton pump inhibitor^†^	0 (0%)	16 (37%)	14 (47%)	12 (60%)	49 (70%)	17 (57%)	<0.0001
Type 2 Diabetes	0 (0%)	22 (51%)	9 (30%)	7 (35%)	23 (33%)	6 (30%)	0.35
MELD score	NA	8.3 ± 2.6	9.7 ± 3.4	12.4 ± 4.5	14.4 ± 4.8	19.8 ± 8.2	<0.0001
Ascites	NA	0 (0%)	0 (0%)	20 (100%)	70 (100%)	23 (77%)	<0.0001
Prior HE	NA	0 (0%)	30 (100%)	0 (0%)	70 (100%)	22 (73%)	<0.0001
Lactulose	NA	0 (0%)	30 (100%)	0 (0%)	70 (100%)	20 (67%)	<0.0001
Rifaximin^†^	NA	0 (0%)	16 (53%)	0 (0%)	41 (59%)	14 (48%)	<0.0001
SBP prophylaxis	NA	0 (0%)	0 (0%)	1 (5%)	6 (9%)	7 (23%)	<0.001
Alcohol-related etiology^†^	NA	7 (16%)	8 (27%)	6 (30%)	35 (50%)	13 (43%)	<0.0001
Daily caloric intake	2229 ± 239	2120 ± 421	2094 ± 406	2010 ± 522	2151 ± 510	1973 ± 910	0.09
Hospitalizations in 90 days^	0 (0%)	2 (5%)	2 (10%)	6 (30%)	34 (48%)	21 (70%)	<0.0001
Death in 1 year^	0 (0%)	0 (0%)	0(0%)	0(0%)	14 (20%)	14 (47%)	0.006

^ higher in infected compared to decompensated patients, five patients in the infected group died during the hospitalization. ^†^ Higher in infected patients (n = 30) compared to all decompensated patients (n = 120)

Of the 30 patients with infections, 19 had SBP, and 11 had UTIs. The diagnosis of SBP was made using PMN count of the ascitic fluid in all patients, and of these, 13 had no growth in ascitic fluid culture while the remaining had *Streptococcus* spp in two, and one each of unknown gram-positive rods, *Candida, Enterococcus faecalis* and *Citrobacter freundii*. Of the 11 UTIs, six grew *E. coli*, four had *Enterococcus* spp grown, and one was *Klebsiella* in the urine.

#### Patients with cirrhosis had higher virulence factor abundance than controls:

As expected, there was a higher abundance of most pathobionts and lower autochthonous taxa in cirrhosis compared to controls was accompanied by a greater abundance of VFs across several species and mechanisms. As shown in Figure 2, the highest fold changes were with Enterobacteriaceae components, with most VFs related to attachment and siderophores rather than exolysins. LPS, as expected, was also higher in cirrhosis than controls (Table S1).

#### Virulence factor abundances are higher in more advanced compared to compensated patients:

In Table 3 and figures S1A-E, when patients with cirrhosis, including those with infections, using FDR-adjusted Kruskal–Wallis analysis, specific VFs that were higher in the top 20 differentiators were attachment machinery (fimbriae), siderophore (salmochelin, aerobactin, enterobactin, and pyoverdine), LPS and one secreted factor pul (pneumolysin). Most factors were consistent between the decompensated groups but distinct from those found in the compensated patients (Table S2). When we only focused on the six pathogens (Tables S3/S4), most VFs that differentiated between subgroups were again siderophores, motility, and attachment factors belonging to *E. coli* and *Pseudomonas. E. faecalis* did not show differences between groups, and minor changes were seen with respect to *Staphylococcus* and *Streptococcus* spp. These patterns were largely consistent regardless of whether infected patients were included or not.Table 2

**Table 2.** Top 20 Virulence Factors on FDR-Adjusted Kruskal–Wallis Comparisons

Compensated (n = 43)	Ascites only (n = 20)	HE only (n = 30)	Both Ascites+HE (n = 70)	Infected patients (n = 30)
ABC transporter	AEC (SS184)	***Aerobactin (CVF852)***	***Aerobactin (CVF852)***	***Aerobactin (CVF852)***
AEC (SS184)	***Aerobactin (CVF852)***	Capsule (VF0323)	E.coli laminin-binding fimbriae (ELF) (CVF824)	Cell wall associated fibronectin binding protein (CVF107)
Alginate (VF0091)	***colibactin (TX033)***	CupE fimbriae (AI449)	Ent (VF0562)	Cytadherence organella (CVF574)
Catalase (CVF760)	***Colibactin (VF0573)***	direct heme uptake system (IA046)	***Ent siderophore (CVF849)***	Ent (VF0562)
***colibactin (TX033)***	E.coli laminin-binding fimbriae (ELF) (CVF824)	E.coli laminin-binding fimbriae (ELF) (CVF824)	Glutamine synthesis (CVF311)	***Ent siderophore (CVF849)***
***Colibactin (VF0573)***	EaeH (CVF679)	ESX-3 (T7SS) (CVF635)	Leucine synthesis (CVF309)	LPS (VF0085)
direct heme uptake system (IA046)	Ent (VF0562)	Leucine synthesis (CVF309)	Methionine sulfoxide reductase (CVF762)	Methionine sulfoxide reductase (CVF762)
ESX-1 (T7SS) (CVF298)	Enteroaggregative immunoglobulin repeat protein (CVF739)	LOS (CVF396)	P216 (CVF580)	Opacity protein (CVF192)
Heme uptake (CVF460)	ESX-1 (T7SS) (CVF298)	Lysine synthesis (CVF310)	peritrichous flagella (AI139)	P146 (CVF579)
LOS (CVF396)	LPS (VF0085)	Methionine sulfoxide reductase (CVF762)	Proteasome-associated proteins (CVF656)	P216 (CVF580)
ND (AI144)	ND (AI144)	P216 (CVF580)	pseudomonine (IA004)	**pul (SS215)**
Polar flagella (VF0473)	***pyoverdine (IA001)***	Proteasome-associated proteins (CVF656)	**pul (SS215)**	RcsAB (VF0571)
PrrA/B (CVF332)	RcsAB (VF0571)	***pyoverdine (IA001)***	***pyoverdine (IA001)***	**Salmochelin (CVF850)**
***Pyochelin (CVF553*)**	Sal (VF0563)	***Salmochelin (CVF850)***	RegX3 (CVF667)	Streptococcal collagen-like proteins (CVF116)
SenX3 (CVF666)	***Salmochelin (CVF850*)**	Stc (CVF021)	***Salmochelin (CVF850)***	T6SS-III (CVF862)
T3SS (SS039)	Stg fimbriae (AI047)	Streptococcal collagen-like proteins (CVF116)	T6SS (SS193)	Type 3 fimbriae (CVF848)
Tap type IV pili (CVF783)	T6SS-III (CVF862)	T3SS (SS039)	T6SS (SS194)	Type 3 fimbriae (VF0567)
Type III secretion system (CVF374)	Type 3 fimbriae (VF0567)	T6SS (CVF782)	T6SS-III (CVF862)	Type I fimbriae (CVF847)
VapA (SS095)	Type I fimbriae (VF0566)	T6SS (SS194)	Type 3 fimbriae (CVF848)	Type I fimbriae (VF0566)
***Yersiniabactin (VF0136)***	Type III secretion system (CVF374)	Type III secretion system (CVF374)	Type I fimbriae (CVF847)	Vlh/pMGA (CVF596)

In this analysis unadjusted for clinical factors, siderophores are prominent in decompensated patients (in bold text and italics)

**Table 3.** MAAslin2 Analysis of Hospitalizations and death in patients without infections

Hospitalizations	Direction	p-value	q-value
**MELD score**	**↑**	3.16E-09	3.24E-06
AIDA I type (*Enterobacteriaceae diffuse adhesin*)^13^	**↑**	3.68E-08	1.88E-05
Outer membrane protein (*Gram-negative bacteria diffuse adhesin*)^14^	**↑**	2.74E-07	9.35E-05
Hyaluronate lyase_(*Streptococcus/Staphylococcus*)^15^	**↑**	3.07E-06	0.000786
Thermostable hemolysin (*Vibrio parahemolyticus*)^16^	**↑**	6.48E-06	0.001328
SigA (*Enterobacteriaceae soluble immunoglobulin A protease*)^17^	**↑**	1.94E-05	0.003306
EaaA (ESBL production)^18^	**↑**	3.87E-05	0.005178
Peg (*Enterobacteriaceae inner membrane transporter*)^19^	**↑**	4.04E-05	0.005178
EaaC_(ESBL production)	**↑**	8.83E-05	0.00891
Pet,_SPATE (*Enterobacteriaceae serine protease*)^20^	**↑**	9.56E-05	0.00891
TSS_1_(*Staphylococcus secretion system*)*^21^	**↓**	0.000148	0.01261
**Hepatic Encephalopathy**	**↑**	0.000231	0.016926
**On lactulose**	**↑**	0.000225	0.016926
OapA (*Hemophilus adhesin*)^22^	**↑**	0.000248	0.016934
MsrAB_(*Staphylococcus methionine sulfoxide reductase*)^23^	**↑**	0.000758	0.040892
SCI_(Salmonella_centrosome_island)(*Salmonella*)^24^	**↑**	0.000719	0.040892
T6SS_1_(Commensal communications)*^25^	**↓**	0.000723	0.040892
**Death**	**Direction**	p-value	q-value
Type I pili_(*Uropathogenic E. Coli*)^26^	**↑**	9.57E-06	0.009
VirB/VirD4_type_IV secretion system Beps_(*Bartonella*)^27^	**↑**	1.74E-05	0.009
**MELD score**	**↑**	3.18E-05	0.01
CDT (*C. difficile*)^28^	**↑**	4.04E-05	0.01
LepA (*Pseudomonas protease*)^29^	**↑**	6.76E-05	0.01
ETT2 (*E. Coli*)*^30^	**↓**	0.0001	0.02
Alkaline protease (*Pseudomonas*)^31^	**↑**	0.0003	0.03
CupB fimbriae (*Uropathogenic E. Coli*)^14^	**↑**	0.0003	0.03
S-layer protein (*Gram-negatives*)^32^	**↑**	0.0003	0.03
Phospholipase D (*A. baumanii, K. pneumoniae*)^33^	**↑**	0.0003	0.03
Opacity protein (*Streptococcus*)^34^	**↑**	0.0005	0.04

* Associated with lower risk of the outcome; rest are associated with higher risk of outcomes, Bold are clinical factors, in parentheses are the origin and potential function of the virulence factors, ↑: associated with outcome, ↓: protective against the outcome.

Figure 3 shows the comparison of bacterial species and VFs between compensated and decompensated patients with cirrhosis. Higher relative abundance of oral taxa such as *Veillonella* spp, *Lactobacillus* spp, and Enterobacteriaceae members were seen in decompensated patients, while Bacteroides and Clostridium spp were higher in compensated patients. Following this, decompensated patients had higher fold changes of several VFs belonging to biofilm (Esp, EcbA) and attachment factors (fimbriae, pili, fibronectin, and fibrinogen-binding proteins). Few siderophores were higher in compensated patients, but this was below the two log2fold change limit.

Comparing those on rifaximin versus those who were not, we found higher abundance of potential SCFA-producing taxa belonging to Lachnospiraceae and Ruminococcaceae in those without rifaximin since these patients are not as advanced in their liver disease. These changes in bacterial species were maintained on MAAslin2. On the other hand, rifaximin therapy was associated with a reduction in VFs in those on rifaximin despite them being at a higher disease severity than those who were not and two VFs, Catalase and Colibactin, were higher in rifaximin non-users on MAAslin2 analysis (Tables S9-10 and Figure S4).

### Hospitalizations were higher in advanced patients and linked with higher VFs independent of clinical factors and infections:

In uninfected patients, 44 were hospitalized over 90 days. The major reasons were hepatic encephalopathy (n = 19) followed by acute kidney injury (AKI, n = 7), infection (n = 11), GI bleeding (n = 3), and liver-unrelated causes (n = 9). Infections were precipitants of HE in five cases and independent of HE in the remaining six. Of the 11 infections, Methicillin-resistant *Staphylococcus aureus* (MRSA) was found in three patients (2 bacteremia and one SBP), *Candida* in three patients (*C. parapsilopsis* in one and *C. albicans* in two), *Streptococcus viridians* bacteremia in one patient, and no organism isolated in four patients (cellulitis and pneumonia in two each). Three patients had two infections during the same hospitalization (SBP followed by UTI). In those with infections, five died during the index admission, and of those discharged alive, readmissions within 90 days were seen in 21 patients, of whom 13 were admitted for HE, 5 for AKI, and 3 for ascites. In addition, three patients with HE also had infections (Candidemia, MRSA, and VRE, one each).

As shown in Figure 4a, on unadjusted DESeq2 analyses, those without hospitalizations had higher autochthonous taxa while *Lactobacillus* and *Veillonella* spp were higher in those who got hospitalized. The findings from the unadjusted data were confirmed on MAAslin2 using only bacterial species (Figures S2A/B, Table S5). Also, lactulose use, HE, MELD, the composite endpoint of cirrhosis subgroups, were associated with higher hospitalizations. We performed multivariable analysis for VFs, including patients with or without infections. When we analyzed MAAsLin2 in those without infections, as expected MELD score, HE and lactulose were associated with hospitalizations, but multiple VFs belonging to ESBL production, soluble factors against immunoglobulin A, and biofilm formation were higher while only an older T6SS system was associated with lower hospitalization. This trend continued when infected patients were added, where despite controlling for MELD score, HE, lactulose, and composite clinical class, there was a major linkage between 90-day hospitalizations and biofilm and attachment VFs (Figure 4b). These patterns were largely replicated when the six major pathogens were studied (Table S7).

### Deaths at one year were associated with higher VF abundance independent of clinical factors and infections:

In patients without infections, 14 patients died over one year; all of them had prior HE, and seven were on rifaximin therapy. All deaths occurred due to liver-related reasons; infections leading to ACLF in 10, massive variceal bleeding in 2, and the remaining two died of hepatocellular cancer complications. Fourteen of the 30 infected patients died: 5 during the index hospitalization due to ACLF, the remaining due to ACLF (n = 6), alcohol-related hepatitis (n = 2), and one due to hepatocellular cancer.

Figure 5a shows on DESeq2, pathobionts belonging to *Pseudomonas, Campylobacter*, and ammonia-producing *Streptococcus* spp, and most Lactobacillus spp were higher in those who died. At the same time, *Lachnospira, Anaerostipes*, and Bifidobacteria spp were lower. On MaAsLin2, patients who died had a lower abundance of autochthonous and short-chain fatty acid-producing taxa, and a higher abundance of potential pathobionts despite controlling for cirrhosis subgroups MELD, HE, and lactulose use (Figure S3A/B, Table S6). As shown in Table 3, in those without infections, VFs that enhance toxigenicity (*C. difficile, Pseudomonas*), enhance attachment (fimbriae, pili), and biofilm formation were higher in those who died. ETT2 from *E. coli* was the only VF protective against death. In those with infections also, Table 4 and Figure 5b again showed several VFs that aided in biofilm formation, adherence, and secretion systems independent of MELD, HE, lactulose use, and composite endpoint, were associated with death. Similar patterns were seen when the six major pathogens were studied (Table S7).

**Table 4.** Hospitalizations and Death according to Virulence Factor patterns using MaAsLin2 including those with infections

Hospitalizations	Direction	p-value	q-value
**MELD score**	**↑**	9.58E-13	1.19E-09
**Composite endpoint**	**↑**	1.68E-10	1.04E-07
**Hepatic Encephalopathy**	**↑**	7.67E-07	3.18E-04
**Lactulose use**	**↑**	2.78E-06	8.63E-04
Esp *(Enterococcus surface protein)^35^*	**↑**	1.13E-04	0.023013
Atl (*Staphylococcus aureus autolysin*)^36^	**↑**	8.33E-04	0.063363
SigA (*Enterobacteriaceae soluble immunoglobulin A protease*)^17^	**↑**	9.95E-04	0.068778
Cell wall hydrolase	**↑**	0.001363	0.078888
AIDA.I.type *(Enterobacteriaceae diffuse adhesin)^13^*	**↑**	0.001459	0.078888
Cry5Aa *(Bacillus thuringensis)^37^*	**↑**	0.001582	0.078888
PilA.type.pili.PGS1 pilin.gene.clusters.1*(E. Coli and Salmonella)*	**↑**	0.001839	0.078888
Lipoprotein diacylglyceryl transferase *(E. Coli)^38^*	**↑**	0.002191	0.089534
BopD (*Enterococcus biofilm*)^39^	**↑**	0.002285	0.089534
Cell wall-associated fibronectin binding protein (*Staphylococcus*)^40^	**↑**	0.002347	0.089534
Fibronectin binding protein (*Staphylococcus*)^40^	**↑**	0.002447	0.089534
SgrA (*Enterococcus biofilm*)^41^	**↑**	0.002744	0.094806
**Death**	**Direction**	**p-value**	**q-value**
**Composite endpoint**	**↑**	4.08E-08	5.08E-05
**MELD score**	**↑**	1.49E-07	9.30E-05
VirB VirD4 type IV secretion system … translocated.effector.Beps. (*Bartonella*)^27^	**↑**	3.41E-05	0.008849
**Lactulose use**	**↑**	3.56E-05	0.008849
**Hepatic Encephalopathy**	**↑**	5.31E-05	0.011005
P216.CVF580. *(Mycoplasma pneumoniae)^42^*	**↑**	8.69E-05	0.013514
Cytadherence.organella.CVF574. *(Mycoplasma pneumoniae)^43^*	**↑**	1.17E-04	0.016106
SgrA (*Enterococcus biofilm*)^41^	**↑**	0.001137	0.05472
Opacity.protein *(Opacity proteins Neisseria)^44^*	**↑**	0.001144	0.05472
Cry5Aa. *(Bacillus thuringensis)^37^*	**↑**	0.001301	0.057431
BopD (*Enterococcus biofilm*)^39^	**↑**	0.001393	0.057431
P97.P102.paralog.family *(Mycoplasma hypopneumoniae)^45^*	**↑**	0.001438	0.057431
YapK.CVF869. (*Yersinia pestis autotransporter*)^46^	**↑**	0.00157	0.057431
Transferrin.binding.protein.2 (*Neisseria*)^47^	**↑**	0.001703	0.058837
LisR.LisK.CVF253. (*Listeria*)^48^	**↑**	0.002083	0.063638
EcbA (*Enterococcus biofilm*)^41^	**↑**	0.002097	0.063638
LepA (*Pseudomonas protease*)^29^	**↑**	0.002587	0.073617

Composite endpoint is a score of decompensation: 0 = compensated, 1 = HE only, 2 = Ascites only, 3 = both HE and ascites, 4 = infected, in parentheses are the origin, and potential function of the virulence factors, Bold are clinical factors, ↑: associated with outcome

#### Capsule FMT RCT:

We included 20 patients on lactulose, rifaximin, and PPI whose baseline and 30-day visit data are shown in tables S8/9. There were no safety signals as previously published regarding the FMT or placebo within four weeks (Table S9).^49^ No significant changes in MELD score, cirrhosis complications, or infections were seen in either group. FMT was associated with a significant reduction in Zn-metalloprotease, T6SS_II, and Capsule VF0144. (Figure 6a). In Figure 6b, Zn-metalloprotease and PhoP/R were the only ones higher in post-FMT vs. post-placebo, while several other VFs such as enterobactin, pili synthesis, T6SS secretion systems (Sci-1 from enteroaggregative *E. coli*), and chaperonins (GroEL hsp60) were higher in post-placebo patients. Neither of the two VFs higher post-FMT was of donor origin since they were not present in the donor (Figure 6c).

## Discussion:

Several prior studies have found a relative increase in abundance of potentially pathogenic taxa belonging to Enterobacteriaceae with worsening disease severity in cirrhosis. However, some of these taxa are also known to be commensals in the gut whose mere presence should not indicate a poor prognosis.^50^ Our study shows that VF analysis results are different from those obtained by evaluating the abundance of microbiota. We also found that VFs in pathogens that enhance iron metabolism, adhesion, and biofilm formation are higher in those with greater cirrhosis severity and prior complications. Selected virulence factors associated with death and hospitalizations independent of clinical biomarkers and are reduced after FMT.

As our understanding of the gut-liver axis changes in cirrhosis matures, studies are beginning to focus on what microbiota are actually doing rather than their mere presence^1^. This includes specific bacterial metabolites as well as genes that signify pathogenicity through the promotion of antibiotic resistance or the ability to destroy host tissues and form biofilms. Traditional pathogenic factors such as LPS have been extensively studied in cirrhosis.^6^ However, the armamentarium of virulence factors is multi-dimensional, extending onto secretion systems, siderophores, biofilm and attachment processes, and secreted lysins that span gram-negative and gram-positive organisms. The data regarding the abundance of these genes are not traditionally reported in metagenomic outputs and could have a bearing on disease progression.

We found multiple microbial virulence factors that were associated with negative outcomes in patients with cirrhosis. These factors originate from several species, some of which are not usual pathogens, and on the other hand, several species that are higher in advanced patients are not represented by VFs. These patterns were also similar in those with clinically apparent infections. These findings are interesting because, in addition to the prior studies showing altered metabolomics and antibiotic resistance genes, the higher prevalence of VF-containing bacteria likely reflect a hostile gut microbiome in cirrhosis.^51,52^

An interesting finding was the relatively higher abundance of siderophores that were present in decompensated cirrhosis even after FDR correction. Siderophores are secondary metabolites that are typically involved in iron metabolism and transport, but of late their communication and virulence potential has been described.^53^ Our findings of pyoverdine, enterobactin, aerobactin, and salmochelin that were higher in more advanced disease likely means that the taxa associated with their production, which are predominantly gram-negative organisms, could potentially worsen with disease.^54–60^ It is interesting though that although higher as disease progressed, siderophores did not significantly contribute toward death and hospitalizations when clinical biomarkers were taken into consideration. This indicates that siderophore abundance could be a co-variate of cirrhosis severity rather than independently contributory.

In contrast, VFs related to the formation of biofilms and attachment proteins were associated with prediction of death and hospitalizations independent of clinical biomarkers. Most of these VFs were encoded by Enterobacteriaceae members, *Staphylococcus*, and *Enterococcus* spp, which are known pathogens.^13,14,26,36,39,41,61^ Biofilms are complicated communities which can protect bacteria from host immune cells and can resist clearance.^62^ These have been shown to be impaired in several GI diseases including inflammatory bowel disease and can worsen mucus degradation.^63–65^ Given the major role for bacterial translocation in patients with cirrhosis, the bacterial biofilm may be relevant in these patients. We also found several attachment strategies (pili, fimbriae, adhesins, and fibronectin proteins, with details in Tables 3 and 4) that help microbiota attach to host tissues which were independently related to outcomes.^66^ These included attachment proteins from uropathogenic *E. coli* even when uninfected patients were studied.^14^ However, not all VFs were associated with higher risk of outcomes, with those belonging to secretion systems that can also be used by commensals or those that have undergone evolutionary attrition at lower risk.^25,30^ This highlights the need to further separate the mere presence of pathobionts from those that could be markers of future negative outcomes likely through virulence factors.

The multiplicity of these species with their virulence factors demonstrate that it is unlikely that only one microbe is associated with cirrhosis progression. Rather a guild or multiplicity of taxa and their virulence factors are likely involved. An interesting study on reducing cytolysin-producing *Enterococcus faecalis* in animal models of alcohol-related liver disease showed benefit.^67^ We did not find that cytolysin production was different between groups or independently predictive of outcomes in cirrhosis. Moreover, even when restricted to six prominent pathogens, none of the VFs originating from *E. faecalis* were associated with differences, and the one associated with outcomes was associated with biofilm formation.^61^ This could be due to a higher association of cytolysin-producing *E. faecalis* with alcoholic hepatitis or due to the use of false discovery correction in the presence of the other VFs. Therefore, we may need to determine the impact of replacing or enhancing entire communities on outcomes in future studies in human cirrhosis.

Replacing the hostile microbiota with those enriched in autochthonous taxa, such as with FMT, could potentially alleviate this burden.^68^ This was shown in findings of our small trial of oral FMT where, in addition to reducing the abundances of potentially pathogenic taxa belonging to Enterobacteriaceae as a whole, the virulence factor-enriched pathogens were also reduced. We found a relative reduction in gram-negative bacterial VFs such as siderophores (enterobactin), adhesion VFs (pili synthesis factors), T6SS secretion systems (Sci-1 from enteroaggregative *E. coli*), and chaperonins (GroEL hsp60) post-FMT compared to post-placebo.^26,55,69,70^ The only two VFs higher post-FMT compared to post-placebo, Zn-metalloprotease, and PhoP/R, were not donor-related, originate from *Mycobacterium* spp, and are unlikely to be of clinical significance in the US population.^71^ However, although FMT can also be a source of drug-resistant and virulence-factor carrying bacteria,^72^ we found none of the VFs were donor-derived.^73^ Adequate donor selection is critical to not only reduce pathobionts but, therefore, also to reduce virulence factor carriage.^74^ The FMT data in cirrhosis are in contrast to prior probiotic and prebiotic studies where the clinical and microbiological evidence basis is not as strong, and the VF dynamics have not been analyzed in detail.^6^ We studied capsules, but other modalities of FMT delivery may yield different results and need to be compared in the future.

Whether these VF-expressing bacteria are a marker of poor prognosis, such as those with antibiotic resistance genes or whether they result in clinically apparent infections is an important question.^52^ Therefore, we enrolled hospitalized patients with SBP and UTI, which are the most common infections in cirrhosis.^5^ Reflecting clinical experience, the yield of causative organisms in UTI was higher than that in SBP on routine culture, and these organisms were consistent with recent literature.^5^ However, there was a disconnect between VF presence and potential causative organisms since most infected patients had higher VF expressions across several bacterial species that reflected underlying liver disease progression. An imbalance between VF-expressing bacteria, local and systemic immune response, therefore, maybe more important in determining individual susceptibility to infections rather than either alone.^75^ This imbalance could potentially explain why SBP is mono-microbial despite the gut microbiome being enriched in multiple different taxa.^76^ Therefore, as the multiple reasons for death and hospitalization over the follow-up period indicate, microbiota enriched in VFs were associated with cirrhosis progression as a whole and not just infections.

The implications of these results are potentially to consider VF abundances rather than simple taxonomic composition to refine prognostication in infected and uninfected patients with cirrhosis. This is illustrated by *Lactobacillus* spp that was higher in advanced cirrhosis, likely as an epiphenomenon of lactulose use but were not associated with VF production.^77^ This point was further shown by the multiple autochthonous taxa that were lower in those on rifaximin on multi-variable analysis but the opposite impact of rifaximin was seen on VFs. This extends a prior rifaximin study and reiterates the need to determine more than simple presence of the microbiota.^78^ Our findings build upon the traditional metagenomic output toward VF-producing targetable taxa. Several of these secreted VFs or their genes could be potential targets of small molecules or phage therapy that can focus the field from antibiotics and FMT to precision therapies.^67,79^ With further validation, major VFs from Enterobacteriaceae and *Enterococcus* spp responsible for biofilm and adhesins could be potentially developed as a rapid qPCR panel. Finally, the small FMT trial provides a proof-of-concept that a substantial change in the microbiome may be needed to affect VF carriage in cirrhosis, and further clinical trials using FMT or other microbiota-based therapeutics are needed to optimize the potential of this approach.

The current study is limited by the use of VF gene abundances but not the actual quantification of the gene products using qPCR, protein quantitation, mRNA changes, or functional assays. We also included a relatively modest sample size of infected inpatients that could be exposed to a different environment than outpatients, but care was taken to collect samples before antibiotic therapy. Data regarding VFs and hospitalizations and death are associations without any causal inferences. Our study is also limited by the cross-sectional analysis of the largest group of patients. Multiple, longitudinal assessments could have enhanced our understanding of VFs evolution over time.

We conclude that in a cohort of patients with cirrhosis with and without clinically apparent infections, virulence factors focused on attachment proteins and biofilm formation are higher in decompensated cirrhosis patients and are associated with death and hospitalizations independent of clinical factors. These virulence factors span several species raising the possibility that impaired microbial dynamics across several taxa are associated with cirrhosis progression. A proof-of-concept FMT trial demonstrated a reduction in virulence factor burden in decompensated patients. Further studies are needed to determine the impact of targeting multiple bacterial virulence factors in patients with cirrhosis to potentially improve outcomes.

## Patients and methods:

### Subjects:

**Figure 1.**
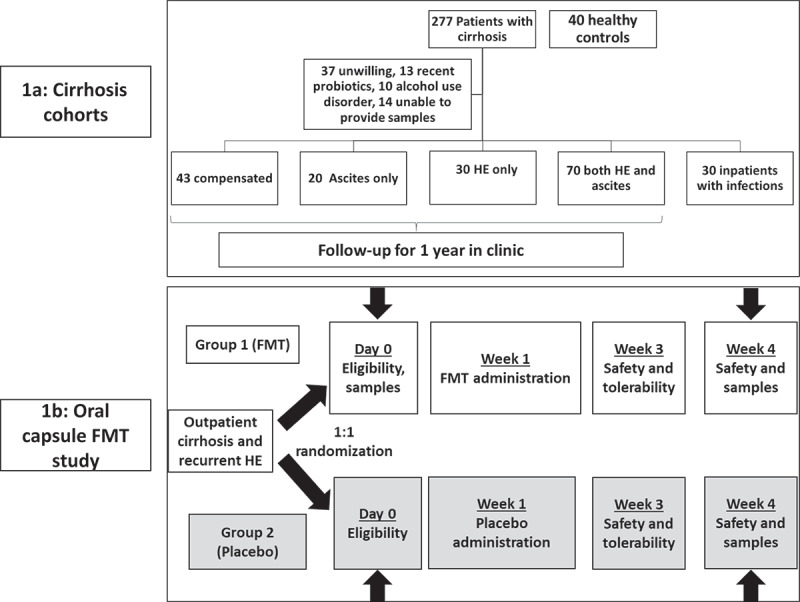
**Schema of both studies** A) Non-trial cohort. B)FMT trial

*Non-trial Cohort*: We enrolled patients with cirrhosis and healthy controls between 21 and 75 years of age after informed consent (Figure 1a). Cirrhosis was defined by liver biopsy, endoscopic or radiological evidence of varices, or porto-systemic shunting in chronic liver disease, frank decompensation, or through transient elastography. Patients unable to provide consent or samples, those with HIV infection, prior transplant, those with alcohol abuse or probiotic use within the prior eight weeks, or those in whom the diagnosis of cirrhosis was unclear were excluded. For outpatients, we also excluded those receiving non-absorbable antibiotics within three months apart from those already on SBP prophylaxis. Patients were divided into compensated (no prior or current history of HE or ascites), ascites only (no HE), HE only (no ascites), both HE and ascites as well, and infected patients. A separate group of inpatients with similar eligibility criteria as above admitted with spontaneous bacterial peritonitis (SBP) or urinary tract infections (UTI) were also enrolled. The stool was collected on admission before antibiotics were started. Healthy controls were recruited through word of mouth or through community advertising. Only individuals free of chronic disease and medications, including PPI (proton pump inhibitors), were considered healthy controls. In addition to fecal samples, data pertaining to cirrhosis severity and concomitant medications were also recorded.

**Figure 2.**
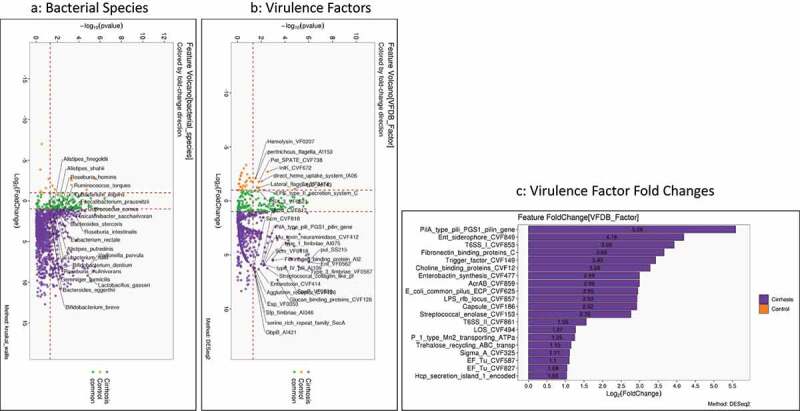
Comparison between healthy controls (n = 40) and all patients with cirrhosis (n = 193). Controls are coded in Orange and cirrhosis in purple A) Kruskal-Wallis comparison of all bacterial species on metagenomics B) DESeq2 comparison of Virulence factors C) Log2fold change on DESeq2 showing the highest virulence factor fold changes in patients with cirrhosis

**Figure 3.**
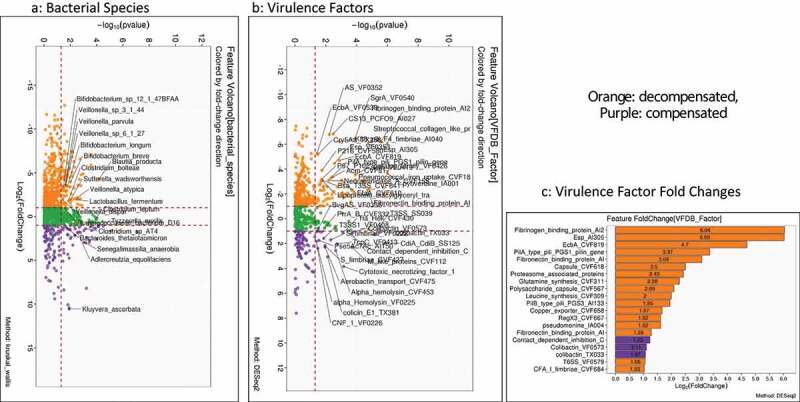
Comparison between compensated and decompensated cirrhosis. Compensated patients are in purple while decompensated patients are in Orange A) Kruskal-Wallis comparison of all bacterial species on metagenomics B) DESeq2 comparison of Virulence factors C) Log2fold change on DESeq2 showing the highest virulence factor fold changes in patients with decompensated cirrhosis

**Figure 4.**
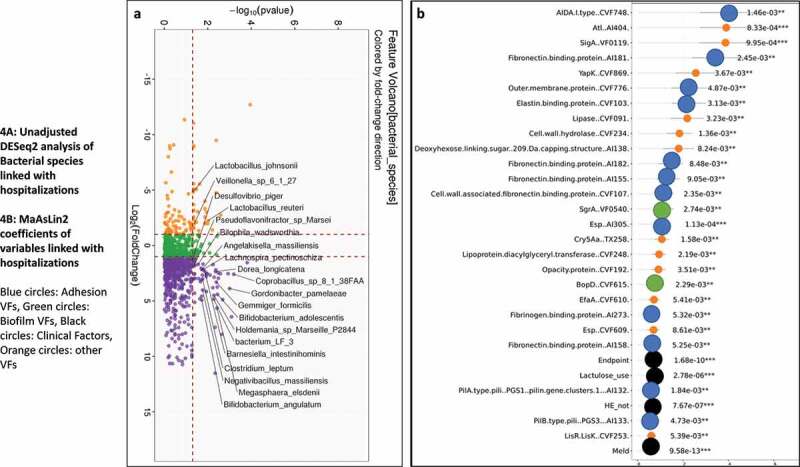
**Comparison of metagenomic bacterial species between those who required hospitalizations (n = 65) compared to those who did not (n = 123) at 90 days**; five patients had died during index admission in the infected group. A) Unadjusted DESeq2 comparison showing organisms with higher fold abundance in those who got hospitalized (Orange) versus those who did not (purple) B) On multi-variable analysis with clinical variable and virulence factors, VFs that were associated with higher hospitalizations are shown in the Cleveland plot. Blue circles: Adhesion VFs, Green circles: Biofilm VFs, Black circles: Clinical Factors, Orange circles: other VFs

**Figure 5.**
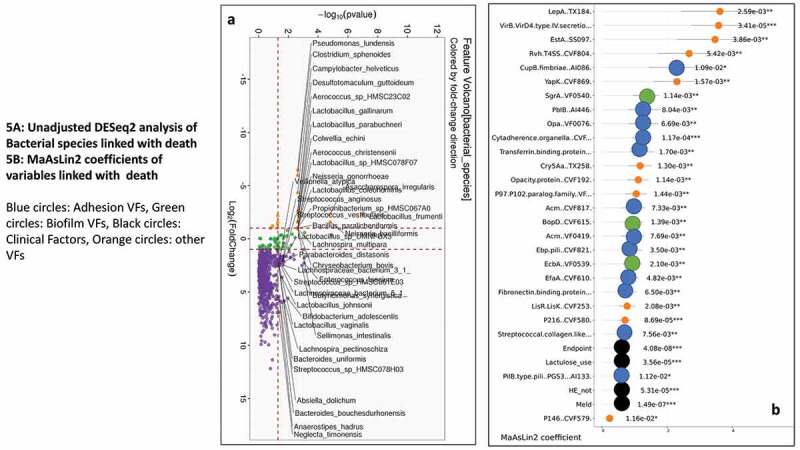
**Comparison of metagenomic bacterial species between those died who required hospitalizations (n = 28) compared to those who did not (n = 165) at one year**. A) Unadjusted DESeq2 comparison showing organisms with higher fold abundance in those who died (Orange) versus those who did not (purple) B) On multi-variable analysis with clinical variable and virulence factors, VFs that were associated with higher rate of death are shown in the Cleveland plot. Blue circles: Adhesion VFs, Green circles: Biofilm VFs, Black circles: Clinical Factors, Orange circles: other VFs

**Figure 6.**
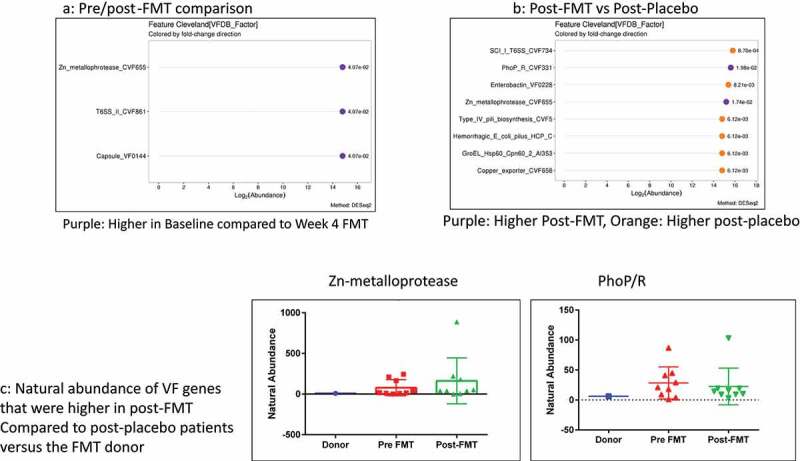
Capsule FMT trial: A) Cleveland plot arranged according to log2 fold abundance and *p*-values between pre (baseline) and post-FMT with purple: Higher in Baseline compared to Week 4 FMT B) Cleveland plot arranged according to log2 fold abundance and *p*-values between post-FMT vs post-placebo with Purple: Higher Post-FMT, Orange: Higher post-placebo C) Natural abundance of VF genes that were higher in post-FMT compared to post-placebo patients versus the FMT donor showing that none of these came from the donor

All patients with cirrhosis were followed for 90 days for non-elective hospitalizations and one year for death risk.

*Capsule FMT trial*: In a previously published randomized clinical trial, outpatients with cirrhosis on rifaximin and lactulose were randomized 1:1 into receiving 15 capsules of FMT from one Openbiome donor selected for the higher relative abundance of Lachnospiraceae and Ruminococcaceae versus 15 capsules of placebo.^49^ The stool was collected pre-intervention and after week 4 (Figure 1b). The clinical trial results have been published as well as 16S rRNA changes.^49^ We analyzed the metagenomic data for VFs pre vs. post-FMT and post-FMT vs. post-placebo and also studied the FMT donor sequences to determine VFs that could have been transmitted to the recipients.

Biorepository: DNA was extracted as below and stored at −80°C as part of a biorepository to which all subjects had consented to participate in. The protocols were approved by the IRBs at the VCU and Richmond VA Medical Centers.

Microbial DNA extraction and sequencing: Metagenomic DNA from fecal samples was extracted using the MO BIO PowerFecal DNA Isolation Kit (Qiagen) and stored in our repository at −80°C until the metagenomics analysis. Samples were processed in an automated, high throughput manner using the QiaCube DNA/RNA Purification System (Qiagen) with bead beating in 0.1 mm glass bead plates. Isolated DNA was quantified and normalized using the Quant-iT Picogreen dsDNA Assay Kit. Shotgun metagenomic libraries were prepared with a procedure adapted from the Nextera Library Prep Kit (Illumina). Libraries were subsequently pooled and assessed using the Agilent Bioanalyzer. Sequencing was performed on either an Illumina NextSeq 550 (1 × 150 bp, NextSeq 500/550 High Output v2 kit) or an Illumina NovaSeq 6000 (1 × 100 bp, NovaSeq 6000 S2 Reagent Kit).

Metagenomic analysis: Reads were processed and annotated using the BoosterShot in-house pipeline. Bcl files were converted to fastq format using bcl2fastq (Illumina). Cutadapt^80^ was used for adapter and quality (final Q-score >20) trimming. Reads shorter than 50 bp were filtered out using cutadapt, and all reads were trimmed to 100 bp before downstream alignment and annotation. Quality sequences were then aligned at 97% identity to a curated database (Venti) containing all representative genomes in RefSeq^81^ for bacteria and additional manually curated strains using the BURST optimal aligner.^82^ Ties in alignment were broken by minimizing the overall number of unique Operational Taxonomic Units (OTUs). For the taxonomic assignment, each input sequence was assigned the lowest common ancestor, which was consistent across at least 80% of all reference sequences tied for the best hit. Counts were normalized to the species-level average genome length. OTUs accounting for less than one-millionth of all species-level genomic markers were discarded, as well as those with either less than 0.01% of their unique genome or less than 1% of the whole genome covered by reads in any sample. Statistical tests were performed with the Wilcoxon signed-rank test with multiple hypothesis correction via false discovery rate (FDR) when applicable. Significance was tested using a *p*-value cutoff of 0.05.

### Analysis of VFs:

We used the Virulence factor database (VFDB), which provides a searchable tool as well as BLAST search of pathogens and VFs for the cross-sectional and trial study results. The metagenomic reads were mapped against the virulence factor database (http://www.mgc.ac.cn/VFs/).^11,83^ This was done using an integrated and automatic pipeline, VFanalyzer, to identify VFs in the bacterial genomes systematically. We also focused on VFs from six major pathogens (*Escherichia, Klebsiella, Enterococcus, Pseudomonas, Streptococcus*, and *Staphylococcus spp)*. For all significant VFs, the functional role and origin were elucidated using published literature review.

### Bio-informatics analysis:

Using BiomMiner^84^ and DESeq2,^85^ we compared patients with cirrhosis to controls, then within cirrhosis with/without decompensation, and finally those who required hospitalizations at 90 days and death in 1 year. To ensure that infected patients did not skew the data, we also performed these analyses without infected patients. We compared bacterial species as well as VFs in the non-trial cohort. Only log2fold change >2 or <-2 with *p* < .05 differences are considered. Kruskal-Wallis with FDR correction was used to evaluate VFs within the cirrhosis groups as a whole and for the six specific pathogens. We also compared bacterial species and VFs in patients with cirrhosis on rifaximin versus those who were not. Then, we performed these analyses usingMAAsLin2 for hospitalization and death for the VF moieties separately, including clinical variables such as age, sex, alcohol etiology, diabetes, PPIs, lactulose, rifaximin, prior HE and MELD score.^86^ For MAAsLin2, we created a composite endpoint (0 = compensated, 1 = HE only, 2 = Ascites only, 3 = both HE and ascites, 4 = infected) within the cirrhosis cohort and included that as an independent variable.

Finally, changes in VFs pre vs. post-FMT and post-FMT vs. post-placebo were performed for the FMT trial. VFs that were increased post-FMT compared to post-placebo or baseline were then investigated within the healthy human donor to determine the source.

## Supplementary Material

Supplemental Material

## Data Availability

Due to individual consent restrictions at VA Medical Centers, metadata cannot be shared. We will upload de-identified sequencing data to a public registry after acceptance and make the SRA IDs available.
