# Supplementary material for: Multiple bacterial virulence factors focused on adherence and biofilm formation associate with outcomes in cirrhosis
Source: Gut Microbes. 2021 Nov 8;13(1):1993584. doi: 10.1080/19490976.2021.1993584 (PMC8582993; doi:10.1080/19490976.2021.1993584)
Supplement: Supplemental Material [file KGMI_A_1993584_SM6599.zip › Supplementary Tables for GUT MICROBES 9_26_21.docx]

**Supplementary Data**

**Table S1: Kruskal-Wallis Adjusted for Virulence Factors across the Non-trial Cohort**

| **Virulence Factor** | **Adjusted p value** | **Controls** | **Compensated** | **Ascites only** | **HE only** | **Ascites+**  **HE** | **Infection** |
| --- | --- | --- | --- | --- | --- | --- | --- |
| GroEL (CVF403) | 1.88E-08 | 0.382552 | 0.118172 | -0.21244 | 5.48E-04 | -0.14211 | -0.54533 |
| Sigma A (CVF325) | 3.17E-08 | 0.244646 | 0.053266 | -0.34386 | 0.111986 | 0.049307 | -0.48423 |
| Trehalose-recycling ABC transporter (CVF651) | 5.36E-08 | 0.223759 | 0.027203 | -0.33965 | 0.132907 | 0.032237 | -0.60947 |
| Alginate biosynthesis (CVF522) | 7.43E-08 | 0.427409 | 0.169608 | -0.52269 | 0.034897 | -0.01007 | -0.97579 |
| Fibronectin-binding protein (AI171) | 1.02E-07 | 0.503993 | 0.081936 | -0.15997 | -0.06054 | -0.08075 | -1.99172 |
| Purine synthesis (CVF306) | 1.02E-07 | 0.609872 | 0.187636 | -0.33826 | -0.01196 | -0.32622 | -2.9144 |
| P(1)-type Mn2+ transporting ATPase (CVF657) | 1.19E-07 | 0.259036 | 0.032539 | -0.46981 | 0.181602 | -0.02316 | -0.88288 |
| Streptococcal collagen-like proteins (CVF116) | 4.24E-07 | 0 | 0 | 0 | 1.744256 | 0 | 2.190502 |
| HSI-3 (SS180) | 5.09E-07 | 0.426544 | 0.07076 | -0.36817 | 0.225584 | -0.06584 | -1.54426 |
| (p)ppGpp synthesis and hydrolysis (CVF335) | 8.30E-07 | 0.273173 | 0.042623 | -0.44748 | 0.134553 | 0.097227 | -0.86693 |
| Copper exporter (CVF658) | 1.04E-06 | 0.237437 | -0.07129 | -0.61769 | 0.304674 | 0.095318 | -1.30995 |
| Accessory secretion factor (CVF299) | 1.12E-06 | 0.566411 | 0.110956 | -0.40905 | 0.086092 | 0 | -1.84196 |
| ACE T6SS (CVF736) | 1.12E-06 | 0.157519 | 0.200322 | 0.271552 | 0.087422 | -0.12184 | -2.65616 |
| LPS O-antigen (P. aeruginosa) (CVF520) | 1.63E-06 | 0.585908 | 0.271695 | -0.30738 | 0.044842 | -0.11745 | -2.90222 |
| HasA-type hemophore-mediated heme uptake system (IA041) | 2.78E-06 | 0.952341 | 0.022916 | -0.37663 | -0.08579 | 0.008399 | -2.65201 |
| Catalase (CVF760) | 2.85E-06 | 0.555779 | 0.457173 | -0.53062 | -0.2291 | -0.40967 | -1.16207 |
| GroEL(Hsp60)/Cpn60.2 (AI353) | 3.55E-06 | 0.233594 | 0.008418 | -0.37561 | 0.170178 | 0.020213 | -0.49804 |
| T6SS-1 (CVF642) | 3.68E-06 | 0.577284 | 0.241897 | -0.33194 | 0.068648 | -0.08635 | -1.27664 |
| Cya (VF0028) | 3.68E-06 | 0.527104 | 0.123118 | -0.32129 | -0.01301 | -0.06953 | -2.92314 |
| EF-Tu (CVF587) | 4.63E-06 | 0.234004 | 0.031165 | -0.1169 | -0.04304 | -0.04226 | -0.32217 |
| PhoP/R (CVF331) | 4.72E-06 | 0.367654 | 0.019023 | -0.62309 | 0.180181 | 0.142951 | -1.80712 |
| GPL locus (CVF650) | 4.89E-06 | 0.338102 | 0.008336 | -0.45773 | -0.00607 | 0.052046 | -0.75573 |
| GacS/GacA two-component system (CVF529) | 4.89E-06 | 0.36258 | 0.032285 | -0.19296 | 0.045869 | -0.15047 | -0.91422 |
| colibactin (TX033) | 5.99E-06 | 1.520584 | 1.237754 | 0.815882 | -1.92879 | -1.15492 | -2.19691 |
| Colibactin (VF0573) | 1.07E-05 | 1.416843 | 1.642916 | 0.661727 | -1.92879 | -1.11168 | -2.2328 |
| MprA/B (CVF333) | 1.11E-05 | 0.45094 | 0.148105 | -0.25488 | 0.046951 | -0.00394 | -2.15325 |
| P216 (CVF580) | 1.39E-05 | -1.92879 | -2.11168 | -1.92879 | 0.749529 | 0.611947 | 1.84193 |
| Capsule (CVF775) | 1.46E-05 | 0.428823 | 0.028796 | -0.17438 | 0.088684 | -0.06303 | -0.82263 |
| Vlh/pMGA (CVF596) | 1.58E-05 | 0 | 0 | 0 | 0 | 0 | 1.833757 |
| ABC transporter (CVF316) | 1.75E-05 | 0.562759 | 0.161776 | -2.5504 | 0.349371 | -0.02292 | -1.98894 |
| ClpC (VF0072) | 1.87E-05 | 0.427638 | 0.112258 | -0.11011 | 0.064998 | -0.13585 | -0.7575 |
| Hemolysin (CVF417) | 1.87E-05 | 0.501984 | 0.057239 | -0.05912 | 0.025143 | -0.10592 | -0.92281 |
| The repeat in toxin (RTX) (CVF795) | 1.87E-05 | 0.295427 | -0.04041 | -0.72349 | 0.228545 | 0.127353 | -1.14595 |
| ABC transporter (CVF516) | 1.87E-05 | 0.91569 | 0.471755 | -1 | -0.48817 | -0.12446 | -1.93983 |
| EF-Tu (CVF827) | 2.95E-05 | 0.189618 | 0.038251 | -0.14092 | 2.02E-04 | -0.00504 | -0.33203 |
| Hcp secretion island-1 encoded type VI secretion system (H-T6SS) (CVF535) | 4.97E-05 | 0.332873 | -0.01334 | -0.36921 | 0.184944 | -0.08668 | -0.74609 |
| Opacity protein (CVF192) | 8.62E-05 | 0 | 0 | 0 | 0 | 0 | 1 |
| Cell wall associated fibronectin binding protein (CVF107) | 1.22E-04 | 0 | 0 | 0 | 0 | 0 | 0.584963 |
| Flagella (CVF521) | 1.22E-04 | 0.323651 | 0.124731 | -0.1855 | -0.00623 | -0.08072 | -0.4591 |
| SenX3 (CVF666) | 1.38E-04 | 2.725612 | 1 | 0 | 0 | 0 | 0 |
| Polar flagella (CVF786) | 1.55E-04 | 0.393295 | 0.182392 | -0.26375 | -0.11367 | -0.02327 | -1.26271 |
| T6SS (SS194) | 1.55E-04 | -1.37014 | -1 | 0 | 2.785502 | 2.15686 | -1.58496 |
| GroEL(Hsp60) (AI317) | 1.82E-04 | 0.507414 | 0.010635 | -0.36069 | -0.32472 | -0.09571 | -0.51137 |
| VapA (SS095) | 2.29E-04 | 1.346724 | 0.939577 | -1.92879 | -0.36639 | -1.17702 | -2.19691 |
| T3SS (SS039) | 2.56E-04 | 0 | 2.448356 | 0 | 1.265243 | 0 | 0 |
| Alginate regulation (CVF523) | 4.12E-04 | 0.233004 | 0.059297 | -0.24918 | 0.125693 | -0.05108 | -0.55485 |
| Glutamine synthesis (CVF311) | 4.21E-04 | 0.16526 | -0.16184 | -0.65424 | 0.416369 | 0.203003 | -0.62772 |
| T2SS (Type II secretion system) (CVF466) | 4.76E-04 | 0.039999 | 0.279563 | 0.140823 | 0.225246 | 0.009624 | -3.04237 |
| RegX3 (CVF667) | 5.21E-04 | 0.072983 | -0.07256 | -0.52616 | 0.263096 | 0.191741 | -1.9343 |
| P146 (CVF579) | 6.24E-04 | 0.102083 | -0.0667 | -0.61679 | 0.034349 | -0.07506 | 0.532216 |
| gsp (SS206) | 7.16E-04 | 0.044835 | 0.232023 | 0.163233 | 0.234917 | -0.01303 | -3.08744 |
| AEC (SS184) | 8.90E-04 | 0 | 2.74911 | 2.31446 | 0 | -0.58496 | -1.37014 |
| Alginate (VF0091) | 0.001135 | 0.924941 | 0.433942 | -2.15686 | 0.428471 | 0.081352 | -2.26524 |
| LOS (CVF396) | 0.001533 | 2.712433 | 1.886027 | 0 | 1.923142 | -1.37014 | -1 |
| E.coli laminin-binding fimbriae (ELF) (CVF824) | 0.001861 | 0 | 0 | 2.086079 | 2.741119 | 1.370143 | -1.37014 |
| Aerobactin (CVF852) | 0.001864 | -2.2328 | -2.15686 | 2.691074 | 1.116436 | 2.391748 | 2.718073 |
| Zn++ metallophrotease (CVF655) | 0.001902 | 0.170207 | 0.018548 | -0.57932 | 0.3401 | 0.096938 | -0.6097 |
| pseudomonine (IA004) | 0.002166 | 0.263742 | 0.026955 | -0.60118 | 0.33282 | 0.149139 | -2.32343 |
| Methionine sulphoxide reductase (CVF762) | 0.002214 | 0 | 0 | 0 | 2.334491 | 1.928789 | 0 |
| T6SS (SS193) | 0.003424 | 0.53426 | 0.123706 | -0.78587 | 0.362026 | 0.222517 | -1.62525 |
| Polar flagella (VF0473) | 0.003496 | 0.754491 | 0.533737 | -0.13674 | 0.198542 | -0.65066 | -1.38805 |
| AdeFGH efflux pump/transport autoinducer (CVF773) | 0.003572 | 0.552228 | 0.256319 | -0.08981 | 0.103214 | -0.27343 | -2.79301 |
| Enteroaggregative immunoglobulin repeat protein (CVF739) | 0.004482 | 0 | 0 | 2.134444 | 0 | 0 | 0 |
| Ent (VF0562) | 0.004793 | -2.15686 | 0.056016 | 1.399845 | 0.223085 | 0.267979 | 1.198021 |
| EF-Tu (VF0460) | 0.004793 | 0.484974 | -0.00755 | -0.39354 | 0.138946 | 0.021939 | -0.98894 |
| CupE fimbriae (AI449) | 0.004992 | 0 | 0 | 0 | 1.995897 | 0 | 0 |
| Enterobactin synthesis (CVF477) | 0.005698 | -0.13203 | 0.119842 | 0.281135 | 0.198933 | -0.12243 | -1.16104 |
| Enterobactin (VF0228) | 0.005995 | -0.04897 | 0.10848 | 0.133965 | 0.081413 | -0.00644 | -0.85485 |
| Listeria adhesion protein (CVF228) | 0.006105 | 0.304232 | -0.01361 | -0.1124 | 0.07992 | -0.03666 | -0.33294 |
| Capsular polysaccharide (CVF282) | 0.006458 | 0.331306 | 0.157978 | -0.25938 | 0.153047 | -0.09587 | -0.67751 |
| Leucine synthesis (CVF309) | 0.006509 | 0.26759 | -0.40182 | -1.49668 | 1.046642 | 0.605939 | -1.95292 |
| Cytadherence organella (CVF574) | 0.006801 | 0 | 0 | 0 | 0 | 0 | 1.321928 |
| Proteasome-associated proteins (CVF656) | 0.007981 | 0.108496 | -0.02335 | -0.63477 | 0.636737 | 0.279135 | -0.84606 |
| T6SS-III (CVF862) | 0.008294 | -2.5624 | -0.13572 | 0.327838 | 0.271944 | 0.19426 | 0.410496 |
| Yersiniabactin siderophore (CVF458) | 0.008428 | -0.0339 | 0.199612 | 0.165972 | -0.10224 | -0.09974 | -1.59521 |
| Elastin binding protein (CVF103) | 0.009274 | 0 | 0 | 0 | 0 | 0 | 0 |
| pul (SS215) | 0.009586 | -2.60584 | -0.29826 | 0.259145 | 0.082413 | 0.235624 | 0.242943 |
| LPS (VF0033) | 0.009913 | 0 | 0 | 0 | 0 | 0 | 0 |
| UpaG adhesin, trimeric AT (CVF740) | 0.010083 | -0.07019 | 0.153777 | 0.288713 | 0.101274 | -0.0674 | -3.11526 |
| pyoverdine (IA001) | 0.0103 | 0 | 0 | 0.584963 | 2.96893 | 1.928789 | 0 |
| EaeH (CVF679) | 0.0103 | -0.18099 | 0.321746 | 0.347221 | 0.192435 | -0.06444 | -3.06002 |
| Stg fimbriae (AI047) | 0.011411 | 0 | 0 | 2.214964 | 0 | 0 | 0 |
| Pyrimidine biosynthesis (CVF845) | 0.013276 | 0.798063 | 0.299771 | -0.4497 | -0.04461 | -0.06107 | -1.0216 |
| peritrichous flagella (AI140) | 0.013546 | -0.0456 | 0.07969 | 0.186684 | 0.077763 | -0.01784 | -0.74515 |
| Type I fimbriae (CVF426) | 0.014365 | -0.11084 | 0.226025 | 0.228391 | 0.14508 | -0.05273 | -1.42329 |
| Type I fimbriae (VF0566) | 0.016935 | 0 | 0 | 2.023128 | 0 | 0 | 0.584963 |
| LOS (CVF494) | 0.018347 | 0.211306 | -0.01046 | -0.17423 | -0.06295 | -0.08227 | -0.27785 |
| Tap type IV pili (CVF783) | 0.018547 | 2.056918 | 2.232796 | 0 | 0 | 0 | 0 |
| Capsule (VF0323) | 0.018794 | 1.244588 | 0.223085 | -0.25047 | 0.72347 | -0.14706 | -2 |
| Hemorrhagic E.coli pilus (HCP) (CVF825) | 0.01883 | -0.12568 | 0.11333 | 0.277939 | 0.174406 | -0.00188 | -1.49699 |
| T2SS (VF0333) | 0.01883 | 0.070155 | 0.31758 | 0.218853 | 0.262582 | -0.05866 | -2.91735 |
| Sal (VF0563) | 0.019184 | 0 | 0 | 0.841958 | 0 | 0 | 0 |
| ESX-3 (T7SS) (CVF635) | 0.020305 | 2.413409 | 0 | 0 | 1.995897 | 0 | 0 |
| Heme uptake (CVF460) | 0.022015 | -0.05403 | 0.384704 | 0.175435 | 0.045906 | -0.24864 | -2.11168 |
| PrrA/B (CVF332) | 0.024516 | 2.735119 | 2.030323 | -1.37014 | -0.78518 | -1.37014 | -1.37014 |
| direct heme uptake system (IA046) | 0.025345 | 1.19018 | 0.988448 | -0.73188 | 0.833698 | -0.28726 | -2.11168 |
| enterobactin (IA019) | 0.026319 | -0.22201 | 0.151684 | 0.254575 | 0.076855 | 0.042735 | -1.36276 |
| Ent siderophore (CVF849) | 0.026679 | -0.33247 | -0.14334 | 0.235097 | 0.134985 | 0.248587 | 0.263236 |
| ESX-1 (T7SS) (CVF298) | 0.027543 | 2.574089 | 1.841958 | 0.584963 | 0 | -1 | -1.37014 |
| peritrichous flagella (AI139) | 0.027864 | -1.27323 | -0.11652 | 0.064469 | 0.165198 | 0.257679 | -0.27902 |
| Type I fimbriae (CVF847) | 0.030241 | -2.5956 | 0.086305 | 0.285548 | 0.072185 | 0.477687 | 0.291247 |
| LPS (VF0085) | 0.0318 | 0 | 0 | 1.788036 | 0 | 0 | 1 |
| Salmochelin (CVF850) | 0.036317 | -1 | 0 | 2.391198 | 2.231481 | 1 | 2.072001 |
| Pyochelin (CVF553) | 0.036317 | 1.473484 | 0.786716 | -1.257 | -1.73202 | -0.47181 | -1.34383 |
| Type III secretion system (CVF374) | 0.036949 | -1.92879 | 1.358591 | 0.501117 | 0.721711 | -0.78804 | -1.92879 |
| Enterobactin transport (CVF478) | 0.039295 | -0.00633 | 0.132707 | 0.303103 | 0.028627 | -0.05525 | -0.62707 |
| ND (AI144) | 0.039606 | 1.268786 | 0.417893 | 0.535179 | -1.58496 | -0.21482 | -1.73202 |
| Type 3 fimbriae (CVF848) | 0.040522 | -2.51107 | -0.46607 | -0.08332 | 0.351488 | 0.479544 | 0.360302 |
| Stc (CVF021) | 0.040824 | 0 | 0 | 0 | 1.111675 | 0 | 0 |
| Yersiniabactin (VF0136) | 0.04187 | 0.053966 | 0.391746 | 0.257756 | -0.10251 | -0.41058 | -2.64338 |
| Type 3 fimbriae (VF0567) | 0.046132 | 0 | 0 | 1.411949 | 0 | 0 | 0.584963 |
| RcsAB (VF0571) | 0.047917 | 0 | 0 | 2.15686 | 0 | 0 | 1.660365 |
| T6SS (CVF782) | 0.048126 | 2.681774 | 0 | 0 | 1.98311 | 0 | 0 |
| Lysine synthesis (CVF310) | 0.04973 | 0 | 0 | 0 | 2.303103 | 0 | 0 |

**Table S2: Kruskal-Wallis Within Cirrhosis group VFs without controls or infected patients**

| **Virulence Factor** | **Adjusted p value** | **Compensated** | **Ascites only** | **HE only** | **Both HE and ascites** |
| --- | --- | --- | --- | --- | --- |
| ACE T6SS (CVF736) | 6.46E-06 | 0.249413 | 0.329021 | 0.137814 | -0.10127 |
| Trehalose-recycling ABC transporter (CVF651) | 2.94E-05 | 0.102741 | -0.29337 | 0.182447 | 0.124211 |
| Copper exporter (CVF658) | 3.53E-05 | 0.013901 | -0.54683 | 0.386265 | 0.200945 |
| Sigma A (CVF325) | 4.59E-05 | 0.121296 | -0.27784 | 0.156435 | 0.117288 |
| P(1)-type Mn2+ transporting ATPase (CVF657) | 4.70E-05 | 0.09524 | -0.42559 | 0.236697 | 0.122952 |
| P216 (CVF580) | 4.70E-05 | -2.32193 | -2.19691 | 0.602471 | 0.464889 |
| Streptococcal collagen-like proteins (CVF116) | 6.06E-05 | 0 | 0 | 1.744256 | 0 |
| Colibactin (VF0573) | 6.66E-05 | 2.642916 | 1.24669 | -1.73202 | -0.58496 |
| (p)ppGpp synthesis and hydrolysis (CVF335) | 1.37E-04 | 0.139692 | -0.35785 | 0.205179 | 0.23061 |
| PhoP/R (CVF331) | 1.88E-04 | 0.14539 | -0.53415 | 0.29436 | 0.377642 |
| colibactin (TX033) | 2.23E-04 | 2.607898 | 1.400844 | -1.58496 | -0.58496 |
| ABC transporter (CVF316) | 2.51E-04 | 0.376815 | -2.4656 | 0.544209 | 0.331155 |
| Purine synthesis (CVF306) | 2.51E-04 | 0.516123 | -0.07841 | 0.26114 | -0.07723 |
| T3SS (SS039) | 3.14E-04 | 2.448356 | 0 | 1.265243 | 0 |
| HSI-3 (SS180) | 3.39E-04 | 0.200172 | -0.26662 | 0.302887 | 0.068697 |
| Alginate biosynthesis (CVF522) | 3.76E-04 | 0.372655 | -0.35819 | 0.128159 | 0.137245 |
| T2SS (Type II secretion system) (CVF466) | 3.76E-04 | 0.340851 | 0.194364 | 0.309209 | 0.041117 |
| T6SS (SS194) | 3.76E-04 | -1.58496 | -1.58496 | 2.785502 | 2.15686 |
| gsp (SS206) | 3.99E-04 | 0.257071 | 0.168315 | 0.260196 | 0.009605 |
| AEC (SS184) | 4.22E-04 | 2.74911 | 2.31446 | 0 | -1.37014 |
| Glutamine synthesis (CVF311) | 4.87E-04 | -0.14623 | -0.64758 | 0.472409 | 0.291851 |
| Vlh/pMGA (CVF596) | 4.87E-04 | 0 | 0 | 0 | 0 |
| Catalase (CVF760) | 5.28E-04 | 0.797446 | -0.4013 | -0.09399 | -0.15357 |
| Cell wall associated fibronectin binding protein (CVF107) | 6.09E-04 | 0 | 0 | 0 | 0 |
| P146 (CVF579) | 6.35E-04 | -0.02795 | -0.57987 | 0.061576 | -0.02398 |
| Cry5Aa (TX258) | 7.07E-04 | 0 | 0 | 0 | 0 |
| T6SS-1 (CVF642) | 7.07E-04 | 0.516281 | -0.16603 | 0.263732 | 0.118312 |
| RegX3 (CVF667) | 7.38E-04 | -0.02918 | -0.48103 | 0.290656 | 0.236046 |
| The repeat in toxin (RTX) (CVF795) | 7.82E-04 | 0.024712 | -0.64694 | 0.348247 | 0.252158 |
| GroEL(Hsp60)/Cpn60.2 (AI353) | 8.92E-04 | 0.09682 | -0.31507 | 0.224852 | 0.117376 |
| E.coli laminin-binding fimbriae (ELF) (CVF824) | 0.001138 | -1.37014 | 2.086079 | 2.741119 | 1.370143 |
| LPS O-antigen (P. aeruginosa) (CVF520) | 0.001138 | 0.597957 | -0.12615 | 0.226851 | 0.104851 |
| MprA/B (CVF333) | 0.001138 | 0.412428 | -0.18438 | 0.141515 | 0.211272 |
| Accessory secretion factor (CVF299) | 0.001809 | 0.360836 | -0.29086 | 0.20679 | 0.161944 |
| Cya (VF0028) | 0.001837 | 0.480249 | -0.072 | 0.214907 | 0.244321 |
| Enteroaggregative immunoglobulin repeat protein (CVF739) | 0.002368 | 0 | 2.134444 | 0 | 0 |
| CupE fimbriae (AI449) | 0.003011 | 0 | 0 | 1.995897 | 0 |
| Enterobactin synthesis (CVF477) | 0.00395 | 0.087754 | 0.264249 | 0.171446 | -0.13289 |
| T6SS (SS193) | 0.004752 | 0.267377 | -0.74984 | 0.488564 | 0.312538 |
| Zn++ metallophrotease (CVF655) | 0.004961 | 0.125572 | -0.57663 | 0.41614 | 0.223958 |
| Enterobactin (VF0228) | 0.00539 | 0.103328 | 0.12174 | 0.068278 | -0.02883 |
| pseudomonine (IA004) | 0.005646 | 0.169305 | -0.51983 | 0.405026 | 0.2456 |
| GroEL (CVF403) | 0.005794 | 0.235047 | -0.07113 | 0.104725 | 0.042327 |
| Yersiniabactin siderophore (CVF458) | 0.006481 | 0.212208 | 0.15025 | -0.11762 | -0.11636 |
| Polar flagella (CVF786) | 0.006916 | 0.296572 | -0.12516 | 0.022564 | 0.1234 |
| Proteasome-associated proteins (CVF656) | 0.007027 | 0.052179 | -0.63477 | 0.717144 | 0.353665 |
| Alginate (VF0091) | 0.007296 | 0.848979 | -2 | 0.772298 | 0.45131 |
| GPL locus (CVF650) | 0.007296 | 0.231636 | -0.35565 | 0.105269 | 0.246534 |
| EaeH (CVF679) | 0.007433 | 0.343941 | 0.347221 | 0.114867 | -0.11707 |
| Stg fimbriae (AI047) | 0.00748 | 0 | 2.214964 | 0 | 0 |
| Fibronectin-binding protein (AI171) | 0.007544 | 0.297709 | 0.054703 | 0.128981 | 0.141075 |
| Leucine synthesis (CVF309) | 0.007971 | -0.26089 | -1.44875 | 1.264706 | 0.859821 |
| UpaG adhesin, trimeric AT (CVF740) | 0.007971 | 0.132906 | 0.278976 | 0.065767 | -0.15027 |
| peritrichous flagella (AI140) | 0.009364 | 0.069154 | 0.174935 | 0.070289 | -0.03876 |
| Flagella (CVF521) | 0.0099 | 0.221282 | -0.13706 | 0.041551 | -0.0052 |
| Type I fimbriae (CVF426) | 0.009965 | 0.226025 | 0.21171 | 0.139787 | -0.09356 |
| ABC transporter (CVF516) | 0.011732 | 0.998468 | -0.57847 | -0.22626 | 0.346769 |
| T2SS (VF0333) | 0.011896 | 0.387747 | 0.250288 | 0.294017 | 0.004972 |
| GacS/GacA two-component system (CVF529) | 0.01255 | 0.189743 | -0.06176 | 0.178175 | 0.027747 |
| Alginate regulation (CVF523) | 0.01268 | 0.155088 | -0.19854 | 0.187472 | 0.040925 |
| Hemorrhagic E.coli pilus (HCP) (CVF825) | 0.01285 | 0.104807 | 0.273636 | 0.158009 | -0.05616 |
| Heme uptake (CVF460) | 0.015258 | 0.436266 | 0.139928 | 0.006353 | -0.3102 |
| Methionine sulphoxide reductase (CVF762) | 0.015258 | 0 | 0 | 2.334491 | 1.928789 |
| pyoverdine (IA001) | 0.021394 | -1.37014 | 0.584963 | 2.96893 | 1.928789 |
| direct heme uptake system (IA046) | 0.02956 | 0.988448 | -0.53511 | 0.833698 | 0 |
| Yersiniabactin (VF0136) | 0.03064 | 0.487201 | 0.295367 | -0.10251 | -0.4348 |
| AdeFGH efflux pump/transport autoinducer (CVF773) | 0.034047 | 0.337372 | 0.031769 | 0.20871 | 0 |
| enterobactin (IA019) | 0.034047 | 0.104748 | 0.243097 | 0.061106 | 0.028241 |
| Enterobactin transport (CVF478) | 0.034047 | 0.144583 | 0.320477 | 0.028627 | -0.05525 |
| Lysine synthesis (CVF310) | 0.042067 | 0 | 0 | 2.303103 | 0 |
| Yersiniabactin (CVF051) | 0.045666 | 0.264805 | 0.247155 | -0.07415 | -0.23937 |
| EHS (SS185) | 0.047872 | 0 | 0 | 2.536964 | 0 |
| Invasion of brain endothelial cells (Ibes) (CVF429) | 0.049662 | 0.127382 | 0.22145 | 0.082129 | -0.05407 |

**Table S3: Virulence Factors Kruskal-Wallis within the cirrhosis group based on the 6 species**

| **Organism** | **Virulence Factor** | **Adjusted P value** | **Compensated** | **Ascites only** | **HE only** | **HE+Ascites** | **Infected** |
| --- | --- | --- | --- | --- | --- | --- | --- |
| *E. Coli* | ACE T6SS (CVF736) | 2.14E-06 | 0.249413 | 0.329021 | 0.137814 | -0.10127 | -2.61507 |
|  | colibactin (TX033) | 3.51E-04 | 2.607898 | 1.400844 | -1.58496 | -0.58496 | -2 |
|  | AEC (SS184) | 5.25E-04 | 2.74911 | 2.31446 | 0 | -1.37014 | -1.37014 |
|  | gsp (SS206) | 5.25E-04 | 0.257071 | 0.168315 | 0.260196 | 0.009605 | -3.10803 |
|  | E.coli laminin-binding fimbriae (ELF) (CVF824) | 0.001701 | -1.37014 | 2.086079 | 2.741119 | 1.370143 | -1.73202 |
|  | Enteroaggregative immunoglobulin repeat protein (CVF739) | 0.003221 | 0 | 2.134444 | 0 | 0 | 0 |
|  | Enterobactin (VF0228) | 0.006164 | 0.103328 | 0.12174 | 0.068278 | -0.02883 | -0.88757 |
|  | Yersiniabactin siderophore (CVF458) | 0.007009 | 0.212208 | 0.15025 | -0.11762 | -0.11636 | -1.60066 |
|  | EaeH (CVF679) | 0.00745 | 0.343941 | 0.347221 | 0.114867 | -0.11707 | -3.08608 |
|  | Stg fimbriae (AI047) | 0.00745 | 0 | 2.214964 | 0 | 0 | 0 |
|  | UpaG adhesin, trimeric AT (CVF740) | 0.00757 | 0.132906 | 0.278976 | 0.065767 | -0.15027 | -3.13336 |
|  | peritrichous flagella (AI140) | 0.008167 | 0.069154 | 0.174935 | 0.070289 | -0.03876 | -0.76016 |
|  | Type I fimbriae (CVF426) | 0.008167 | 0.226025 | 0.21171 | 0.139787 | -0.09356 | -1.46249 |
|  | Hemorrhagic E.coli pilus (HCP) (CVF825) | 0.010547 | 0.104807 | 0.273636 | 0.158009 | -0.05616 | -1.51558 |
|  | Heme uptake (CVF460) | 0.01213 | 0.436266 | 0.139928 | 0.006353 | -0.3102 | -2.15589 |
|  | enterobactin (IA019) | 0.02967 | 0.104748 | 0.243097 | 0.061106 | 0.028241 | -1.37863 |
|  | EHS (SS185) | 0.044651 | 0 | 0 | 2.536964 | 0 | 0 |
|  | Invasion of brain endothelial cells (Ibes) (CVF429) | 0.045713 | 0.127382 | 0.22145 | 0.082129 | -0.05407 | -0.58387 |
|  | ETT2 (SS017) | 0.048437 | 2.138709 | 2.130089 | 2.660365 | -1.5624 | -1.92879 |
| *Pseudo-*  *monas* | **Virulence Factor** | **Adjusted P value** | **Compensated** | **Ascites only** | **HE only** | **HE+Ascites** | **Infected** |
|  | Alginate biosynthesis (CVF522) | 2.77E-04 | 0.372655 | -0.35819 | 0.128159 | 0.137245 | -0.84063 |
|  | HSI-3 (SS180) | 2.77E-04 | 0.200172 | -0.26662 | 0.302887 | 0.068697 | -1.48743 |
|  | LPS O-antigen (P. aeruginosa) (CVF520) | 8.98E-04 | 0.597957 | -0.12615 | 0.226851 | 0.104851 | -2.84989 |
|  | CupE fimbriae (AI449) | 0.002219 | 0 | 0 | 1.995897 | 0 | 0 |
|  | Alginate (VF0091) | 5.37E-03 | 0.848979 | -2 | 0.772298 | 0.45131 | -2.11168 |
|  | Flagella (CVF521) | 0.006774 | 0.221282 | -0.13706 | 0.041551 | -0.0052 | -0.4184 |
|  | Alginate regulation (CVF523) | 0.007341 | 0.155088 | -0.19854 | 0.187472 | 0.040925 | -0.50778 |
|  | GacS/GacA two-component system (CVF529) | 0.007341 | 0.189743 | -0.06176 | 0.178175 | 0.027747 | -0.81556 |
|  | pyoverdine (IA001) | 0.010922 | -1.37014 | 0.584963 | 2.96893 | 1.928789 | -1.37014 |
|  | Hcp secretion island-1 encoded type VI secretion system (H-T6SS) (CVF535) | 0.036626 | 0.146144 | -0.25969 | 0.288544 | 0.052027 | -0.64056 |
|  | HasA-type hemophore-mediated heme uptake system (IA041) | 0.038753 | 0.331655 | -0.1308 | 0.147018 | 0.405577 | -2.43091 |
| *Klebsiella*  *pneumoniae* | **Virulence Factor** | **Adjusted P value** | **Compensated** | **Ascites only** | **HE only** | **HE+Ascites** | **Infected** |
|  | Colibactin (VF0573) | 1.28E-05 | 2.642916 | 1.24669 | -1.73202 | -0.58496 | -2 |
| *Streptococcus* | **Virulence Factor** | **Adjusted P value** | **Compensated** | **Ascites only** | **HE only** | **HE+Ascites** | **Infected** |
|  | Streptococcal collagen-like proteins (CVF116) | 2.74E-05 | 0 | 0 | 1.744256 | 0 | 2.190502 |
| *Staphylo*  *coccus* | **Virulence Factor** | **Adjusted P value** | **Compensated** | **Ascites only** | **HE only** | **HE+Ascites** | **Infected** |
|  | Cell wall associated fibronectin binding protein (CVF107) | 1.80E-04 | 0 | 0 | 0 | 0.00E+00 | 0.584963 |
| *Enterococcus Faecalis* | **Virulence Factor** | **Adjusted P value** | **Compensated** | **Ascites only** | **HE only** | **HE+Ascites** | **Infected** |
|  | None | - | - | - | - | - | - |

**Table S4: Virulence Factors Kruskal-Wallis within the cirrhosis group based on the 6 species excluding the infected patients**

| **Organism** | **Virulence Factor** | **Adjusted P value** | **Compensated** | **Ascites only** | **HE only** | **HE+Ascites** |
| --- | --- | --- | --- | --- | --- | --- |
| ***E. Coli*** | F41 fimbriae (AI041) | 0.025438 | 0 | 0.14661 | 0 | 0.23160 |
|  | locus for diffuse adherence (lda), afimbrial adhesin (AI044) | 0.025438 | 0 | 0.2042 | 0 | 0.1126 |
|  | AIDA-I type (CVF748) | 0.08444 | 0 | 0 | 0 | 0.012 |
|  | AEC (SS184) | 0.146683 | 2.74911 | 0.944316 | -1.84196 | -2.36952 |
|  | F41 fimbriae (AI041) | 0.025438 | 0 | 0 | 0 | 0 |
| ***Pseudo-***  ***monas*** | **Virulence Factor** | **Adjusted P value** | **Compensated** | **Ascites only** | **HE only** | **HE+Ascites** |
|  | CupE fimbriae (AI449) | 0.0362834 | 0 | 0 | 1.995897 | 0 |
|  | pyoverdine (IA001) | 0.0362834 | -1.92879 | -0.78518 | 2.96893 | 1.928789 |
| ***Klebsiella***  ***pneumoniae*** | **Virulence Factor** | **Adjusted P value** | **Compensated** | **Ascites only** | **HE only** | **HE+Ascites** |
|  | Colibactin (VF0573) | 0.049703 | 1.057953 | 0.317901 | -2.11168 | -1.37014 |
| ***Streptococcus*** | **Virulence Factor** | **Adjusted P value** | **Compensated** | **Ascites only** | **HE only** | **HE+Ascites** |
|  | Streptococcal collagen-like proteins (CVF116) | 0.048 | 0 | 0 | 1.744256 | 0 |
| ***Staphylococcus*** | **Virulence Factor** | **Adjusted P value** | **Compensated** | **Ascites only** | **HE only** | **HE+Ascites** |
|  | None |  |  |  |  |  |
| ***Enterococcus faecalis*** | **Virulence Factor** | **Adjusted P value** | **Compensated** | **Ascites only** | **HE only** | **HE+Ascites** |
|  | None | - | - | - | - | - |

**Table S5: Metagenomics MAAsLin2 Bacterial species and Hospitalizations**

| **Feature** | **Linked with Hospitalization?** | **Coefficient** | **P-value** | **Q-value** |
| --- | --- | --- | --- | --- |
| **MELD score** | Yes | 0.472196 | 4.12E-12 | 8.55E-09 |
| **Endpoint** | Yes | 0.667439 | 2.84E-10 | 2.95E-07 |
| Lactobacillus.paracasei | Yes | 3.432115 | 4.04E-08 | 2.80E-05 |
| **Hepatic Encephalopathy** | Yes | 0.622943 | 5.62E-07 | 2.10E-04 |
| Veillonella.sp..6_1_27 | Yes | 2.681863 | 6.07E-07 | 2.10E-04 |
| Bifidobacterium.pseudocatenulatum | Yes | 3.165528 | 7.17E-07 | 2.13E-04 |
| Veillonella.sp..3_1_44 | Yes | 2.718276 | 2.30E-06 | 4.77E-04 |
| Bifidobacterium.kashiwanohense | Yes | 3.320051 | 3.55E-06 | 5.50E-04 |
| Veillonella.parvula | Yes | 2.089505 | 3.60E-06 | 5.50E-04 |
| Bifidobacterium.bifidum | Yes | 2.969019 | 3.79E-06 | 5.50E-04 |
| **Lactulose use** | Yes | 0.634878 | 4.99E-06 | 6.10E-04 |
| Veillonella.atypica | Yes | 2.007796 | 6.30E-06 | 6.83E-04 |
| Lactobacillus.reuteri | Yes | 3.332455 | 7.06E-06 | 6.86E-04 |
| Lactobacillus.plantarum | Yes | 2.737861 | 7.27E-06 | 6.86E-04 |
| Lactobacillus.crispatus | Yes | 2.971043 | 1.25E-05 | 0.001039 |
| Lactobacillus.casei | Yes | 3.00914 | 1.70E-05 | 0.001259 |
| Clostridioides.difficile | Yes | 1.841972 | 2.92E-05 | 0.001903 |
| Bifidobacterium.adolescentis | Yes | 3.129436 | 2.93E-05 | 0.001903 |
| Veillonella.dispar | Yes | 2.057804 | 3.06E-05 | 0.001927 |
| Lactobacillus.vaginalis | Yes | 3.48291 | 3.34E-05 | 0.001966 |
| Streptococcus.mutans | Yes | 2.329721 | 6.54E-05 | 0.003313 |
| Lactobacillus.mucosae | Yes | 3.405158 | 6.78E-05 | 0.003353 |
| Clostridium.innocuum | Yes | 2.221187 | 1.03E-04 | 0.004223 |
| Megasphaera.elsdenii | Yes | 2.742555 | 1.12E-04 | 0.004364 |
| Bifidobacterium.longum | Yes | 1.902834 | 1.16E-04 | 0.004364 |
| Lactobacillus.salivarius | Yes | 2.327424 | 1.20E-04 | 0.004364 |
| Veillonella.tobetsuensis | Yes | 3.186581 | 1.45E-04 | 0.004784 |
| Bifidobacterium.breve | Yes | 2.033097 | 1.48E-04 | 0.004784 |
| Lactobacillus.fermentum | Yes | 2.314773 | 1.50E-04 | 0.004784 |
| Veillonella.sp..S13053.19 | Yes | 2.79902 | 1.82E-04 | 0.005477 |
| Escherichia.coli | Yes | 1.775363 | 1.91E-04 | 0.005477 |
| Lactobacillus.gasseri | Yes | 2.132278 | 2.45E-04 | 0.006369 |
| Lactobacillus_timonensis | Yes | 2.265048 | 2.64E-04 | 0.006682 |
| Streptococcus.parasanguinis | Yes | 1.911405 | 2.83E-04 | 0.007091 |
| Lactobacillus.oris | Yes | 2.373041 | 3.02E-04 | 0.007224 |
| Lactobacillus.delbrueckii | Yes | 2.299711 | 4.39E-04 | 0.009593 |
| Streptococcus.pneumoniae | Yes | 1.675182 | 5.78E-04 | 0.012127 |
| Lactobacillus.acidophilus | Yes | 2.940732 | 6.36E-04 | 0.012962 |
| Bifidobacterium.catenulatum | Yes | 4.167925 | 6.38E-04 | 0.012962 |
| Ruminococcus.gnavus | Yes | 1.592138 | 6.65E-04 | 0.013215 |
| Lactobacillus.paragasseri | Yes | 2.331244 | 6.70E-04 | 0.013215 |
| Salmonella.enterica | Yes | 1.905928 | 6.74E-04 | 0.013215 |
| Erysipelatoclostridium.ramosum | Yes | 2.122807 | 8.30E-04 | 0.015255 |
| Raoultella.ornithinolytica | Yes | 2.26465 | 9.37E-04 | 0.01622 |
| Bifidobacterium.scardovii | Yes | 3.88053 | 9.57E-04 | 0.016364 |
| Enterococcus.faecalis | Yes | 1.653699 | 9.97E-04 | 0.016829 |
| Pseudomonas.aeruginosa | Yes | 2.871066 | 0.001049 | 0.017321 |
| Pantoea.sp..PSNIH1 | Yes | 3.618604 | 0.0012 | 0.019026 |
| Klebsiella.pneumoniae | Yes | 1.530534 | 0.001236 | 0.01945 |
| Enterococcus.faecium | Yes | 1.625447 | 0.001296 | 0.019937 |
| Streptococcus.thermophilus | Yes | 1.46674 | 0.001432 | 0.021363 |
| Shigella.flexneri | Yes | 1.949855 | 0.001579 | 0.02326 |
| Veillonella.rogosae | Yes | 3.377965 | 0.001805 | 0.024993 |
| Bacteroides.sp..AF27.33 | Yes | 2.35079 | 0.001926 | 0.026148 |
| Blautia.hansenii | Yes | 1.605706 | 0.001966 | 0.026193 |
| Flavonifractor.plautii | Yes | 1.512177 | 0.002122 | 0.027607 |
| Shigella.sonnei | Yes | 2.146427 | 0.002137 | 0.027607 |
| Clostridium.aldenense | Yes | 1.672097 | 0.002331 | 0.029172 |
| Klebsiella.michiganensis | Yes | 1.828568 | 0.002757 | 0.033391 |
| Clostridium.bolteae | Yes | 1.377365 | 0.003273 | 0.037771 |
| Fusobacterium.nucleatum | Yes | 1.999722 | 0.003297 | 0.037829 |
| Leuconostoc.mesenteroides | Yes | 3.751841 | 0.00338 | 0.038572 |
| Streptococcus.agalactiae | Yes | 2.025333 | 0.003614 | 0.040572 |
| Clostridium.symbiosum | Yes | 1.477689 | 0.003707 | 0.041399 |
| Streptococcus.suis | Yes | 1.289217 | 0.003963 | 0.04355 |
| Faecalibacterium.prausnitzii | Yes | 1.324145 | 0.004656 | 0.048387 |
| Streptococcus.mitis | Yes | 1.975742 | 0.004875 | 0.04988 |
| Lachnoclostridium.sp..YL32 | Yes | 1.416963 | 0.004949 | 0.049925 |
| bacterium_LF.3 | No | 1.426447 | 1.66E-07 | 8.63E-05 |
| Dorea_longicatena | No | 0.582385 | 8.49E-07 | 2.20E-04 |
| Massilioclostridium_coli | No | 0.870568 | 1.17E-06 | 2.70E-04 |
| Angelakisella_massiliensis | No | 0.599928 | 3.35E-06 | 5.50E-04 |
| Clostridium_sp._Marseille.P3244 | No | 1.031198 | 3.97E-06 | 5.50E-04 |
| Bifidobacterium_adolescentis | No | 0.766861 | 4.30E-06 | 5.58E-04 |
| Anaerotruncus_colihominis | No | 0.549999 | 5.40E-06 | 6.23E-04 |
| Dorea_formicigenerans | No | 0.541366 | 6.58E-06 | 6.83E-04 |
| Gemmiger_formicilis | No | 0.610685 | 8.98E-06 | 8.11E-04 |
| Holdemania_sp._Marseille.P2844 | No | 1.108081 | 1.15E-05 | 9.99E-04 |
| Gordonibacter_pamelaeae | No | 1.059167 | 1.53E-05 | 0.001223 |
| Mordavella_sp._Marseille.P3756 | No | 0.674029 | 1.67E-05 | 0.001259 |
| Clostridium_saccharolyticum | No | 0.611814 | 2.01E-05 | 0.00144 |
| Fusicatenibacter_saccharivorans | No | 0.463978 | 2.16E-05 | 0.001492 |
| Agathobaculum_desmolans | No | 0.602514 | 3.38E-05 | 0.001966 |
| Eubacterium_rectale | No | 0.464347 | 3.41E-05 | 0.001966 |
| Holdemania_filiformis | No | 0.671176 | 4.21E-05 | 0.002366 |
| Clostridium_sp._M62.1 | No | 0.517022 | 5.04E-05 | 0.002634 |
| Clostridiales_bacterium_KLE1615 | No | 0.671351 | 5.05E-05 | 0.002634 |
| Coprobacillus_sp._8_1_38FAA | No | 1.312703 | 5.07E-05 | 0.002634 |
| Lachnospira_pectinoschiza | No | 0.648827 | 7.45E-05 | 0.003597 |
| Bilophila_sp._4_1_30 | No | 0.950776 | 7.78E-05 | 0.0036 |
| Blautia_massiliensis | No | 0.405493 | 7.80E-05 | 0.0036 |
| Pseudoflavonifractor_capillosus | No | 0.559767 | 8.09E-05 | 0.003651 |
| Clostridium_sp._ATCC_BAA.442 | No | 0.42567 | 8.37E-05 | 0.003701 |
| Bilophila_wadsworthia | No | 0.778914 | 8.80E-05 | 0.003808 |
| Intestinimonas_massiliensis | No | 0.581656 | 1.03E-04 | 0.004223 |
| Eubacterium_ramulus | No | 0.456346 | 1.04E-04 | 0.004223 |
| Blautia_hansenii | No | 0.58485 | 1.13E-04 | 0.004364 |
| Ruminococcus_champanellensis | No | 1.083496 | 1.14E-04 | 0.004364 |
| Ruminococcus_torques | No | 0.42985 | 1.20E-04 | 0.004364 |
| Roseburia_faecis | No | 0.55785 | 1.22E-04 | 0.004368 |
| Faecalitalea_cylindroides | No | 0.529471 | 1.24E-04 | 0.004368 |
| Erysipelotrichaceae_bacterium_2_2_44A | No | 0.533036 | 1.45E-04 | 0.004784 |
| Ruminococcus_bromii | No | 1.193394 | 1.47E-04 | 0.004784 |
| Lachnospiraceae_bacterium_A2 | No | 0.749819 | 1.70E-04 | 0.005358 |
| Faecalibacterium_prausnitzii | No | 0.359652 | 1.80E-04 | 0.005477 |
| Provencibacterium_massiliense | No | 0.768056 | 1.84E-04 | 0.005477 |
| Blautia_sp._Marseille.P3087 | No | 0.572571 | 1.87E-04 | 0.005477 |
| Bifidobacterium_angulatum | No | 1.130662 | 1.88E-04 | 0.005477 |
| Flavonifractor_plautii | No | 0.372292 | 1.92E-04 | 0.005477 |
| Roseburia_intestinalis | No | 0.45971 | 1.98E-04 | 0.005569 |
| Absiella_dolichum | No | 0.613187 | 2.05E-04 | 0.005667 |
| Bacteroides_pectinophilus | No | 0.529013 | 2.26E-04 | 0.006012 |
| Lachnoanaerobaculum_sp._OBRC5.5 | No | 0.933539 | 2.26E-04 | 0.006012 |
| Megasphaera_massiliensis | No | 1.221431 | 2.28E-04 | 0.006012 |
| Subdoligranulum_variabile | No | 0.490193 | 2.29E-04 | 0.006012 |
| Eubacterium_sp._YI | No | 0.980304 | 2.62E-04 | 0.006682 |
| Clostridium_innocuum | No | 0.512697 | 2.98E-04 | 0.007224 |
| Ruminococcaceae_bacterium_D5 | No | 0.896138 | 2.99E-04 | 0.007224 |
| Erysipelotrichaceae_bacterium_6_1_45 | No | 0.4878 | 3.03E-04 | 0.007224 |
| Alistipes_ihumii | No | 1.141605 | 3.24E-04 | 0.007644 |
| Bacteroides_stercoris | No | 0.401646 | 3.57E-04 | 0.008323 |
| Phocea_massiliensis | No | 0.601963 | 3.78E-04 | 0.008717 |
| Negativibacillus_massiliensis | No | 0.646551 | 3.85E-04 | 0.008769 |
| Clostridium_sp._ATCC_29733 | No | 0.562196 | 3.88E-04 | 0.008769 |
| Bifidobacterium_pseudocatenulatum | No | 0.612812 | 4.17E-04 | 0.009303 |
| Clostridium_phoceensis | No | 0.495339 | 4.29E-04 | 0.009473 |
| Ileibacterium_massiliense | No | 0.928731 | 4.63E-04 | 0.010022 |
| Merdibacter_massiliensis | No | 0.579784 | 4.89E-04 | 0.010477 |
| Coprococcus_comes | No | 0.410213 | 5.55E-04 | 0.011758 |
| Bacteroides_sp._3_1_40A | No | 0.438202 | 5.84E-04 | 0.01213 |
| Subdoligranulum_sp._4_3_54A2FAA | No | 0.441934 | 6.43E-04 | 0.012962 |
| Ruminococcaceae_bacterium_D16 | No | 0.412299 | 6.94E-04 | 0.013364 |
| Bacteroides_uniformis | No | 0.33705 | 6.95E-04 | 0.013364 |
| Clostridium_leptum | No | 0.525662 | 7.22E-04 | 0.013749 |
| Eubacterium_ventriosum | No | 0.493284 | 7.37E-04 | 0.013918 |
| Eubacterium_eligens | No | 0.45308 | 7.46E-04 | 0.01396 |
| Clostridium_sp._L2.50 | No | 0.570989 | 8.23E-04 | 0.015255 |
| Butyricicoccus_pullicaecorum | No | 0.596334 | 8.77E-04 | 0.015919 |
| Ruminococcus_gnavus | No | 0.347807 | 8.84E-04 | 0.015919 |
| Eubacterium_hallii | No | 0.423252 | 8.89E-04 | 0.015919 |
| Eubacterium_siraeum | No | 0.517334 | 9.06E-04 | 0.016083 |
| Anaerotruncus_rubiinfantis | No | 1.308192 | 9.37E-04 | 0.01622 |
| Clostridium_asparagiforme | No | 0.551803 | 9.37E-04 | 0.01622 |
| Dialister_invisus | No | 0.621951 | 9.61E-04 | 0.016364 |
| Eubacterium_limosum | No | 1.189432 | 0.001025 | 0.017177 |
| Ruminococcus_faecis | No | 0.46598 | 0.001058 | 0.017321 |
| Eisenbergiella_massiliensis | No | 0.467537 | 0.001059 | 0.017321 |
| Gordonibacter_urolithinfaciens | No | 1.068138 | 0.00107 | 0.017369 |
| Faecalitalea_sp._Marseille.P3755 | No | 0.498204 | 0.001154 | 0.018453 |
| Blautia_obeum | No | 0.340983 | 0.001155 | 0.018453 |
| Blautia_sp._KLE_1732 | No | 0.461439 | 0.001275 | 0.019874 |
| Coprococcus_eutactus | No | 0.457302 | 0.001282 | 0.019874 |
| Collinsella_bouchesdurhonensis | No | 1.043191 | 0.001308 | 0.019972 |
| Clostridium_saccharogumia | No | 0.643276 | 0.001349 | 0.020446 |
| Marvinbryantia_formatexigens | No | 0.547571 | 0.001426 | 0.021363 |
| Bifidobacterium_catenulatum | No | 0.62342 | 0.00144 | 0.021363 |
| Erysipelotrichaceae_bacterium_21_3 | No | 0.659129 | 0.001593 | 0.023299 |
| Bifidobacterium_kashiwanohense | No | 0.594871 | 0.00162 | 0.023536 |
| Neglecta_timonensis | No | 0.571935 | 0.001635 | 0.023587 |
| Pseudoflavonifractor_sp._Marseille.P3106 | No | 0.444448 | 0.001651 | 0.023655 |
| Emergencia_timonensis | No | 0.611398 | 0.00169 | 0.023819 |
| Lactococcus_lactis | No | 0.508042 | 0.001694 | 0.023819 |
| Roseburia_hominis | No | 0.444152 | 0.001697 | 0.023819 |
| Anaerotruncus_sp._G32012 | No | 0.584989 | 0.001756 | 0.024476 |
| Anaeromassilibacillus_sp._Marseille.P3371 | No | 0.467652 | 0.001841 | 0.025329 |
| Eubacteriaceae_bacterium_CHKCI004 | No | 0.614825 | 0.001869 | 0.025533 |
| Bifidobacterium_longum | No | 0.349563 | 0.001954 | 0.026193 |
| Collinsella_aerofaciens | No | 0.40888 | 0.001967 | 0.026193 |
| Blautia_hydrogenotrophica | No | 0.667752 | 0.00201 | 0.026595 |
| Butyrivibrio_crossotus | No | 0.518235 | 0.002027 | 0.026649 |
| Clostridium_sp._AT4 | No | 0.482918 | 0.00214 | 0.027607 |
| Ruthenibacterium_lactatiformans | No | 0.398013 | 0.002177 | 0.027908 |
| Bittarella_massiliensis | No | 0.674894 | 0.002195 | 0.027975 |
| Alistipes_indistinctus | No | 1.409462 | 0.002235 | 0.028307 |
| Lachnospiraceae_bacterium_3_1_46FAA | No | 0.481336 | 0.002276 | 0.02865 |
| Collinsella_sp._TF06.26 | No | 0.536636 | 0.002347 | 0.029191 |
| Catenibacterium_mitsuokai | No | 0.509736 | 0.002438 | 0.030141 |
| Hespellia_stercorisuis | No | 0.632372 | 0.002622 | 0.032222 |
| Traorella_massiliensis | No | 0.499611 | 0.002639 | 0.032243 |
| Roseburia_inulinivorans | No | 0.349438 | 0.002787 | 0.033391 |
| Bifidobacterium_bifidum | No | 0.483249 | 0.002794 | 0.033391 |
| Bifidobacterium_breve | No | 0.401037 | 0.0028 | 0.033391 |
| Bacteroides_vulgatus | No | 0.295148 | 0.002813 | 0.033391 |
| Lachnoclostridium_phocaeense | No | 0.598844 | 0.002938 | 0.034499 |
| Blautia_sp._SF.50 | No | 0.39734 | 0.00294 | 0.034499 |
| Lachnospiraceae_bacterium_7_1_58FAA | No | 0.36381 | 0.00309 | 0.036054 |
| Odoribacter_splanchnicus | No | 0.49701 | 0.003247 | 0.037675 |
| Actinomyces_bouchesdurhonensis | No | 1.316895 | 0.00342 | 0.038813 |
| Alistipes_shahii | No | 0.453746 | 0.003511 | 0.039631 |
| Parabacteroides_sp._D13 | No | 0.453133 | 0.003767 | 0.041837 |
| Bacteroides_cellulosilyticus | No | 0.438104 | 0.003937 | 0.043501 |
| Lachnospiraceae_bacterium_TF01.11 | No | 0.553759 | 0.003996 | 0.043687 |
| Coprococcus_sp._HPP0074 | No | 0.524451 | 0.00411 | 0.044694 |
| Actinomyces_odontolyticus | No | 0.605172 | 0.004263 | 0.046113 |
| Ruminococcus_bicirculans | No | 0.610252 | 0.004347 | 0.046777 |
| Lactobacillus_rogosae | No | 0.554522 | 0.0044 | 0.04711 |
| Eubacterium_sp._3_1_31 | No | 0.534199 | 0.004482 | 0.047743 |
| Lachnospiraceae_bacterium_3_1_57FAA_CT1 | No | 0.557863 | 0.004626 | 0.048387 |
| Bacteroides_caccae | No | 0.341371 | 0.00464 | 0.048387 |
| Acidaminococcus_intestini | No | 0.727395 | 0.004655 | 0.048387 |
| Ruminococcus_sp._AT10 | No | 0.584329 | 0.004663 | 0.048387 |
| Clostridium_glycyrrhizinilyticum | No | 0.403238 | 0.004683 | 0.048387 |
| Ruminococcus_flavefaciens | No | 1.974273 | 0.004767 | 0.049016 |
| Lachnospiraceae_bacterium_1_1_57FAA | No | 1.213252 | 0.004903 | 0.049924 |
| Johnsonella_ignava | No | 0.572133 | 0.004952 | 0.049925 |

Endpoint: Composite score of decompensation: 0=compensated, 1=HE only, 2=Ascites only, 3=both HE and ascites, 4=infected

**Table S6: Metagenomics MAAsLin2 Bacterial species and Death**

| **Feature** | **Linked with Death?** | **Coefficient** | **P-value** | **Q-value** |
| --- | --- | --- | --- | --- |
| **Endpoint** | Yes | 0.665619 | 4.80E-08 | 9.97E-05 |
| **MELD score** | Yes | 0.495216 | 1.54E-07 | 1.60E-04 |
| **Hepatic encephalopathy** | Yes | 0.597591 | 3.72E-05 | 0.004175 |
| Clostridium.sp..OM04.12AA | Yes | 2.938833 | 5.52E-05 | 0.004568 |
| **Lactulose use** | Yes | 0.629829 | 9.25E-05 | 0.005916 |
| Barnesiella.viscericola | Yes | 2.060039 | 1.05E-04 | 0.005916 |
| Blautia.hansenii | Yes | 1.887235 | 1.19E-04 | 0.005916 |
| Bacteroides.fragilis | Yes | 1.819096 | 1.45E-04 | 0.006165 |
| Chitinophaga.sp..K20C18050901 | Yes | 3.160729 | 1.65E-04 | 0.006573 |
| Streptococcus.gallolyticus | Yes | 2.254134 | 1.96E-04 | 0.007266 |
| Enterococcus.faecium | Yes | 1.724282 | 2.09E-04 | 0.007633 |
| Pantoea.sp..PSNIH1 | Yes | 2.406763 | 2.20E-04 | 0.007755 |
| Clostridium.sp..AM32.2 | Yes | 2.836111 | 2.36E-04 | 0.008042 |
| Clostridium.sp..AM22.11AC | Yes | 2.313947 | 2.48E-04 | 0.008184 |
| Bacteroides.togonis | Yes | 2.171947 | 2.59E-04 | 0.00842 |
| Enterococcus.faecalis | Yes | 1.714568 | 3.18E-04 | 0.009859 |
| Klebsiella.pneumoniae | Yes | 1.587725 | 3.51E-04 | 0.01041 |
| Butyricimonas.sp..H184 | Yes | 1.879894 | 4.80E-04 | 0.012976 |
| Parabacteroides.sp..CT06 | Yes | 1.768652 | 4.81E-04 | 0.012976 |
| Streptococcus.pasteurianus | Yes | 2.119287 | 5.29E-04 | 0.013525 |
| Parabacteroides.distasonis | Yes | 1.720423 | 5.35E-04 | 0.013525 |
| Clostridium.symbiosum | Yes | 1.680653 | 5.45E-04 | 0.013525 |
| Faecalibacterium.prausnitzii | Yes | 1.540603 | 5.59E-04 | 0.013525 |
| Veillonella.parvula | Yes | 1.513313 | 5.90E-04 | 0.014078 |
| Lactobacillus.plantarum | Yes | 1.861069 | 6.42E-04 | 0.014623 |
| Megasphaera.hexanoica | Yes | 2.085789 | 6.55E-04 | 0.014623 |
| Odoribacter.laneus | Yes | 2.276028 | 7.84E-04 | 0.016045 |
| Bacteroides.vulgatus | Yes | 1.606873 | 9.13E-04 | 0.017896 |
| Streptococcus.suis | Yes | 1.486325 | 9.54E-04 | 0.018177 |
| Clostridium.sp..TM06.18 | Yes | 2.449507 | 0.001067 | 0.018985 |
| Enterobacter.asburiae | Yes | 2.620905 | 0.00107 | 0.018985 |
| Klebsiella.michiganensis | Yes | 1.768379 | 0.001079 | 0.018985 |
| Alistipes.sp..An116 | Yes | 2.894201 | 0.001123 | 0.019157 |
| Butyricimonas.faecihominis | Yes | 2.031269 | 0.001205 | 0.019845 |
| Streptococcus.pneumoniae | Yes | 1.54615 | 0.001217 | 0.019845 |
| Bacteroides.massiliensis | Yes | 2.136756 | 0.001219 | 0.019845 |
| Bacteroides.caecimuris | Yes | 1.656749 | 0.001237 | 0.019845 |
| Megasphaera.massiliensis | Yes | 3.898049 | 0.001242 | 0.019845 |
| Bacteroides.ilei | Yes | 2.507406 | 0.001308 | 0.020141 |
| Sutterella.wadsworthensis | Yes | 3.621625 | 0.001409 | 0.021262 |
| Bacteroides.thetaiotaomicron | Yes | 1.640721 | 0.001499 | 0.022033 |
| Bacteroides.faecis | Yes | 1.74071 | 0.001614 | 0.023283 |
| Dorea.formicigenerans | Yes | 1.726623 | 0.001654 | 0.023464 |
| Slackia.isoflavoniconvertens | Yes | 3.63821 | 0.001661 | 0.023464 |
| Bifidobacterium.longum | Yes | 1.663664 | 0.00169 | 0.023578 |
| Pseudomonas_lundensis | Yes | 2.942643 | 0.001722 | 0.023578 |
| Lactobacillus.paracasei | Yes | 1.849966 | 0.001726 | 0.023578 |
| Bacteroides.uniformis | Yes | 1.649717 | 0.00181 | 0.024228 |
| Lactobacillus.fermentum | Yes | 1.607368 | 0.001833 | 0.024254 |
| Megasphaera.elsdenii | Yes | 2.068061 | 0.001896 | 0.024925 |
| Butyricimonas.virosa | Yes | 1.899066 | 0.002155 | 0.026984 |
| Neisseria_bacilliformis | Yes | 2.115287 | 0.002232 | 0.027429 |
| Bacteroides.xylanisolvens | Yes | 1.649323 | 0.002326 | 0.028089 |
| Enterobacter.roggenkampii | Yes | 2.205319 | 0.002402 | 0.028467 |
| Prevotella.disiens | Yes | 2.129669 | 0.002442 | 0.028467 |
| Bacteroides.sartorii | Yes | 1.943958 | 0.002455 | 0.028467 |
| Streptococcus.thermophilus | Yes | 1.415642 | 0.002467 | 0.028467 |
| Klebsiella.quasipneumoniae | Yes | 1.678767 | 0.002503 | 0.028559 |
| Enterococcus.durans | Yes | 1.893126 | 0.002529 | 0.028691 |
| Parabacteroides.sp..20_3 | Yes | 2.04795 | 0.002599 | 0.028905 |
| Bacteroides.sp..4_3_47FAA | Yes | 2.098216 | 0.002683 | 0.029235 |
| Veillonella.dispar | Yes | 1.446191 | 0.002688 | 0.029235 |
| Odoribacter.splanchnicus | Yes | 1.807881 | 0.002824 | 0.030078 |
| Bacteroides.sp..1_1_30 | Yes | 1.954271 | 0.002927 | 0.030554 |
| Escherichia.coli | Yes | 1.431793 | 0.002953 | 0.030668 |
| Bacteroides.dorei | Yes | 1.561429 | 0.003329 | 0.032773 |
| Clostridium.sp..AM25.23AC | Yes | 3.290444 | 0.003437 | 0.033668 |
| Bacteroides.stercoris | Yes | 1.669989 | 0.003486 | 0.033988 |
| Ruminococcus.torques | Yes | 1.463927 | 0.003547 | 0.0342 |
| Klebsiella.variicola | Yes | 1.69779 | 0.003567 | 0.0342 |
| Faecalibacterium.sp..OF03.6AC | Yes | 2.310601 | 0.003575 | 0.0342 |
| Bacteroides.finegoldii | Yes | 1.576093 | 0.00359 | 0.0342 |
| Gabonia.massiliensis | Yes | 2.77778 | 0.003928 | 0.037028 |
| Bacteroides.caccae | Yes | 1.590691 | 0.00394 | 0.037028 |
| Bacteroides.ovatus | Yes | 1.509001 | 0.004057 | 0.037961 |
| Lactobacillus.casei | Yes | 1.78806 | 0.004342 | 0.039826 |
| Prevotella.sp..Marseille.P8229 | Yes | 2.941283 | 0.004458 | 0.040306 |
| Lactobacillus.gasseri | Yes | 1.560814 | 0.004556 | 0.040846 |
| Bacteroides.nordii | Yes | 1.871265 | 0.004563 | 0.040846 |
| Prevotellamassilia.timonensis | Yes | 2.161385 | 0.00464 | 0.041307 |
| Lactobacillus.oris | Yes | 1.591404 | 0.004695 | 0.041307 |
| Veillonella.atypica | Yes | 1.295371 | 0.004743 | 0.041307 |
| Bacteroides.plebeius | Yes | 1.655609 | 0.004753 | 0.041307 |
| Bacteroides.eggerthii | Yes | 1.63399 | 0.004788 | 0.041342 |
| Clostridium.sp..AM30.24 | Yes | 2.191832 | 0.004814 | 0.041342 |
| Alistipes.finegoldii | Yes | 1.612762 | 0.004887 | 0.041565 |
| Bacteroides.cellulosilyticus | Yes | 1.514689 | 0.004943 | 0.041565 |
| Bacteroides.sp..AM16.13 | Yes | 1.812485 | 0.005083 | 0.042232 |
| Bacteroides.mediterraneensis | Yes | 2.311629 | 0.005129 | 0.042439 |
| Alistipes.shahii | Yes | 1.567088 | 0.00521 | 0.042944 |
| Enterobacter.cloacae | Yes | 1.879897 | 0.005278 | 0.043326 |
| Blautia.sp..N6H1.15 | Yes | 1.376809 | 0.005335 | 0.043625 |
| Lactobacillus_parabuchneri | Yes | 1.906453 | 0.005412 | 0.044083 |
| Roseburia.hominis | Yes | 1.435145 | 0.005546 | 0.044825 |
| Parabacteroides.merdae | Yes | 1.757596 | 0.005769 | 0.045906 |
| Enterobacter.hormaechei | Yes | 1.711522 | 0.005861 | 0.046283 |
| Prevotella.lascolaii | Yes | 2.351263 | 0.006041 | 0.047171 |
| Clostridium.bolteae | Yes | 1.343111 | 0.0061 | 0.047276 |
| Ruminococcus.gnavus | Yes | 1.29517 | 0.006173 | 0.047662 |
| Collinsella.tanakaei | Yes | 3.620052 | 0.006221 | 0.047741 |
| Klebsiella.oxytoca | Yes | 1.48972 | 0.006398 | 0.048413 |
| Bacteroides.sp..AM25.34 | Yes | 2.215433 | 0.006522 | 0.048906 |
| Fusicatenibacter_saccharivorans | No | 0.824082 | 2.85E-06 | 0.00162 |
| Clostridium_sp._M62.1 | No | 1.048986 | 3.12E-06 | 0.00162 |
| Ruminococcus_torques | No | 0.804587 | 8.22E-06 | 0.003377 |
| Coprococcus_comes | No | 0.859872 | 9.76E-06 | 0.003377 |
| Dorea_longicatena | No | 0.796134 | 1.51E-05 | 0.00368 |
| Flavonifractor_plautii | No | 0.679319 | 1.75E-05 | 0.00368 |
| Massilioclostridium_coli | No | 1.426688 | 2.05E-05 | 0.00368 |
| Blautia_sp._SF.50 | No | 0.998343 | 2.28E-05 | 0.00368 |
| Dorea_formicigenerans | No | 0.795491 | 2.33E-05 | 0.00368 |
| Anaerotruncus_colihominis | No | 0.838557 | 2.44E-05 | 0.00368 |
| Blautia_massiliensis | No | 0.682935 | 2.54E-05 | 0.00368 |
| Clostridium_sp._ATCC_BAA.442 | No | 0.730693 | 2.66E-05 | 0.00368 |
| Eubacterium_ramulus | No | 0.799874 | 3.08E-05 | 0.003786 |
| Ruminococcaceae_bacterium_D16 | No | 0.833392 | 3.10E-05 | 0.003786 |
| Anaerostipes_hadrus | No | 0.892372 | 4.08E-05 | 0.004175 |
| Eubacterium_rectale | No | 0.725197 | 4.17E-05 | 0.004175 |
| Clostridium_saccharolyticum | No | 1.020519 | 4.22E-05 | 0.004175 |
| Bifidobacterium_longum | No | 0.741497 | 4.88E-05 | 0.004568 |
| Alistipes_putredinis | No | 0.974492 | 5.21E-05 | 0.004568 |
| Angelakisella_massiliensis | No | 0.854649 | 5.38E-05 | 0.004568 |
| Blautia_sp._KLE_1732 | No | 1.004466 | 5.86E-05 | 0.004568 |
| Subdoligranulum_variabile | No | 0.901593 | 5.94E-05 | 0.004568 |
| Pseudoflavonifractor_capillosus | No | 0.948495 | 6.37E-05 | 0.004687 |
| Lachnospira_pectinoschiza | No | 1.128402 | 6.54E-05 | 0.004687 |
| Blautia_obeum | No | 0.666442 | 6.91E-05 | 0.004781 |
| Eubacterium_ventriosum | No | 0.988166 | 8.18E-05 | 0.00548 |
| Bacteroides_bouchesdurhonensis | No | 1.060499 | 9.71E-05 | 0.005916 |
| Mordavella_sp._Marseille.P3756 | No | 1.038771 | 1.02E-04 | 0.005916 |
| Ruminococcus_faecis | No | 0.900599 | 1.04E-04 | 0.005916 |
| Clostridium_sp._AT4 | No | 1.047069 | 1.09E-04 | 0.005916 |
| Faecalibacterium_prausnitzii | No | 0.571996 | 1.11E-04 | 0.005916 |
| Subdoligranulum_sp._4_3_54A2FAA | No | 0.822046 | 1.15E-04 | 0.005916 |
| Eisenbergiella_massiliensis | No | 0.934989 | 1.16E-04 | 0.005916 |
| Absiella_dolichum | No | 1.101178 | 1.22E-04 | 0.005916 |
| Bifidobacterium_adolescentis | No | 1.06337 | 1.22E-04 | 0.005916 |
| Clostridium_phoceensis | No | 0.877768 | 1.28E-04 | 0.006025 |
| Bacteroides_pectinophilus | No | 0.915184 | 1.34E-04 | 0.006165 |
| Gemmiger_formicilis | No | 0.830429 | 1.38E-04 | 0.006165 |
| Roseburia_intestinalis | No | 0.750392 | 1.43E-04 | 0.006165 |
| Neglecta_timonensis | No | 1.211618 | 1.45E-04 | 0.006165 |
| Blautia_wexlerae | No | 0.627727 | 1.51E-04 | 0.006268 |
| Lachnospiraceae_bacterium_5_1_63FAA | No | 1.226891 | 1.55E-04 | 0.006313 |
| Prevotella_copri | No | 1.119728 | 1.76E-04 | 0.006785 |
| Ruthenibacterium_lactatiformans | No | 0.807369 | 1.76E-04 | 0.006785 |
| Holdemania_filiformis | No | 0.995507 | 1.92E-04 | 0.007238 |
| Clostridium_sp._Marseille.P3244 | No | 1.653404 | 2.19E-04 | 0.007755 |
| Roseburia_faecis | No | 0.861479 | 2.24E-04 | 0.00777 |
| Collinsella_aerofaciens | No | 0.780168 | 2.46E-04 | 0.008184 |
| Bacteroides_vulgatus | No | 0.546836 | 3.06E-04 | 0.00964 |
| Eubacterium_eligens | No | 0.756938 | 3.06E-04 | 0.00964 |
| Alistipes_shahii | No | 0.896521 | 3.47E-04 | 0.01041 |
| Ruminococcus_gnavus | No | 0.576795 | 3.47E-04 | 0.01041 |
| bacterium_LF.3 | No | 1.607551 | 3.91E-04 | 0.011431 |
| Parabacteroides_sp._D13 | No | 0.9616 | 4.08E-04 | 0.01176 |
| Bacteroides_dorei | No | 0.648694 | 4.20E-04 | 0.011951 |
| Bacteroides_cellulosilyticus | No | 0.885376 | 4.41E-04 | 0.012389 |
| Butyricicoccus_pullicaecorum | No | 1.099825 | 4.60E-04 | 0.012739 |
| Bacteroides_caccae | No | 0.646438 | 4.92E-04 | 0.013054 |
| Pseudoflavonifractor_sp._Marseille.P3106 | No | 0.815358 | 4.97E-04 | 0.013054 |
| Bacteroides_uniformis | No | 0.515818 | 5.18E-04 | 0.013437 |
| Bifidobacterium_kashiwanohense | No | 1.058683 | 5.53E-04 | 0.013525 |
| Bacteroides_faecichinchillae | No | 1.209627 | 5.60E-04 | 0.013525 |
| Gordonibacter_pamelaeae | No | 1.59467 | 6.01E-04 | 0.014187 |
| Blautia_sp._Marseille.P3087 | No | 0.855818 | 6.27E-04 | 0.014487 |
| Clostridium_sp._SN20 | No | 0.734563 | 6.28E-04 | 0.014487 |
| Phocea_massiliensis | No | 0.994644 | 6.51E-04 | 0.014623 |
| Lachnoclostridium_phocaeense | No | 1.292715 | 6.73E-04 | 0.014874 |
| Johnsonella_ignava | No | 1.321271 | 6.92E-04 | 0.015121 |
| Clostridium_leptum | No | 0.82608 | 7.21E-04 | 0.015563 |
| Lachnospiraceae_bacterium_7_1_58FAA | No | 0.657498 | 7.27E-04 | 0.015563 |
| Blautia_sp._Marseille.P3201T | No | 0.810033 | 7.45E-04 | 0.015794 |
| Bifidobacterium_catenulatum | No | 1.050404 | 7.56E-04 | 0.015863 |
| Collinsella_sp._TF06.26 | No | 0.96656 | 7.72E-04 | 0.016041 |
| Blautia_hansenii | No | 0.817045 | 7.88E-04 | 0.016045 |
| Clostridium_symbiosum | No | 0.590368 | 8.09E-04 | 0.016318 |
| Lactococcus_lactis | No | 0.888501 | 8.30E-04 | 0.016583 |
| Ruminococcaceae_bacterium_cv2 | No | 0.840804 | 8.41E-04 | 0.016632 |
| Roseburia_inulinivorans | No | 0.594982 | 9.37E-04 | 0.018035 |
| Bacteroides_sp._3_1_40A | No | 0.651631 | 9.38E-04 | 0.018035 |
| Collinsella_sp._4_8_47FAA | No | 0.857816 | 9.78E-04 | 0.018459 |
| Catenibacterium_mitsuokai | No | 0.886912 | 9.87E-04 | 0.018469 |
| Faecalitalea_sp._Marseille.P3755 | No | 0.810933 | 9.98E-04 | 0.01851 |
| Odoribacter_splanchnicus | No | 0.896253 | 0.001031 | 0.018857 |
| Parabacteroides_merdae | No | 0.615748 | 0.001035 | 0.018857 |
| Bacteroides_sp._3_1_19 | No | 1.004137 | 0.001054 | 0.018985 |
| Negativibacillus_massiliensis | No | 0.96946 | 0.001098 | 0.019157 |
| Eubacterium_sp._3_1_31 | No | 1.05399 | 0.001122 | 0.019157 |
| Ruminococcus_bicirculans | No | 1.144945 | 0.001125 | 0.019157 |
| Bacteroides_massiliensis | No | 0.668595 | 0.001208 | 0.019845 |
| Agathobaculum_desmolans | No | 0.727005 | 0.001213 | 0.019845 |
| Parabacteroides_sp._D26 | No | 1.017801 | 0.001227 | 0.019845 |
| Bittarella_massiliensis | No | 1.314066 | 0.001299 | 0.020141 |
| Fusicatenibacter_sp._2789STDY5834925 | No | 1.294424 | 0.0013 | 0.020141 |
| Lactobacillus_rogosae | No | 1.07585 | 0.001304 | 0.020141 |
| Acidaminococcus_intestini | No | 1.514059 | 0.001309 | 0.020141 |
| Butyrivibrio_crossotus | No | 0.884326 | 0.001374 | 0.020987 |
| Clostridium_sp._SS2.1 | No | 1.030268 | 0.001413 | 0.021262 |
| Bacteroides_thetaiotaomicron | No | 0.514227 | 0.001433 | 0.021412 |
| Bacteroides_intestinalis | No | 1.143516 | 0.001452 | 0.021545 |
| Fournierella_massiliensis | No | 0.741677 | 0.001506 | 0.022033 |
| Anaerotruncus_sp._G32012 | No | 1.063076 | 0.001577 | 0.022909 |
| Akkermansia_muciniphila | No | 0.724733 | 0.001629 | 0.023331 |
| Barnesiella_intestinihominis | No | 1.218437 | 0.0017 | 0.023578 |
| Intestinimonas_massiliensis | No | 0.718325 | 0.001722 | 0.023578 |
| Alistipes_sp._CHKCI003 | No | 2.096087 | 0.001778 | 0.024139 |
| Blautia_sp._Marseille.P2398 | No | 0.615575 | 0.001812 | 0.024228 |
| Bifidobacterium_breve | No | 0.64164 | 0.00182 | 0.024228 |
| Eubacteriaceae_bacterium_CHKCI004 | No | 1.063207 | 0.00191 | 0.024952 |
| Clostridium_sp._7_3_54FAA | No | 0.682268 | 0.001998 | 0.025933 |
| Streptococcus_salivarius | No | 0.583183 | 0.002066 | 0.026656 |
| Christensenella_minuta | No | 1.105572 | 0.002087 | 0.02676 |
| Butyricimonas_virosa | No | 1.314753 | 0.002111 | 0.026901 |
| Alistipes_finegoldii | No | 0.644196 | 0.002133 | 0.026984 |
| Tyzzerella_nexilis | No | 0.601272 | 0.002157 | 0.026984 |
| Lachnospiraceae_bacterium_TF01.11 | No | 1.001121 | 0.00221 | 0.027325 |
| Hungatella_hathewayi | No | 0.546211 | 0.00221 | 0.027325 |
| Clostridium_sp._KLE_1755 | No | 1.703875 | 0.00228 | 0.027858 |
| Eubacterium_siraeum | No | 0.739543 | 0.002321 | 0.028089 |
| Bacteroides_finegoldii | No | 0.5422 | 0.002355 | 0.028278 |
| Erysipelotrichaceae_bacterium_2_2_44A | No | 0.63089 | 0.002435 | 0.028467 |
| Ruminococcus_lactaris | No | 0.642427 | 0.00245 | 0.028467 |
| Roseburia_hominis | No | 0.65261 | 0.002464 | 0.028467 |
| Bifidobacterium_pseudocatenulatum | No | 0.792237 | 0.002501 | 0.028559 |
| Eggerthella_lenta | No | 0.637438 | 0.002542 | 0.028691 |
| Clostridium_bolteae | No | 0.558913 | 0.002557 | 0.028705 |
| Clostridiales_bacterium_KLE1615 | No | 0.745278 | 0.002606 | 0.028905 |
| Lachnospiraceae_bacterium_6_1_37FAA | No | 1.179259 | 0.002616 | 0.028905 |
| Bifidobacterium_sp._12_1_47BFAA | No | 0.975628 | 0.002636 | 0.02897 |
| Alistipes_senegalensis | No | 0.942815 | 0.002781 | 0.030078 |
| Bacteroides_coprocola | No | 0.755309 | 0.002804 | 0.030078 |
| Alistipes_ihumii | No | 1.627267 | 0.002817 | 0.030078 |
| Gordonibacter_urolithinfaciens | No | 1.916322 | 0.002864 | 0.030353 |
| Bacteroides_sp._HMSC067B03 | No | 0.65449 | 0.002886 | 0.030355 |
| Bacteroides_faecis | No | 1.244295 | 0.002894 | 0.030355 |
| Alistipes_indistinctus | No | 3.513326 | 0.00301 | 0.030967 |
| Bacteroides_sp._2_1_33B | No | 1.134351 | 0.003012 | 0.030967 |
| Intestinimonas_butyriciproducens | No | 0.595469 | 0.003063 | 0.031335 |
| Turicibacter_sanguinis | No | 2.009367 | 0.003091 | 0.031475 |
| Clostridium_innocuum | No | 0.629026 | 0.003119 | 0.031505 |
| Coprococcus_eutactus | No | 0.643905 | 0.003125 | 0.031505 |
| Phascolarctobacterium_succinatutens | No | 1.227136 | 0.003165 | 0.031686 |
| Lachnospiraceae_bacterium_A2 | No | 0.971226 | 0.003183 | 0.031686 |
| Emergencia_timonensis | No | 1.00928 | 0.003192 | 0.031686 |
| Prevotella_bivia | No | 1.43687 | 0.003204 | 0.031686 |
| Bacteroides_sartorii | No | 0.576452 | 0.00358 | 0.0342 |
| Escherichia_coli | No | 0.680484 | 0.003641 | 0.034529 |
| Alistipes_obesi | No | 1.338693 | 0.004146 | 0.038615 |
| Merdibacter_massiliensis | No | 0.738914 | 0.004206 | 0.038999 |
| Lachnospiraceae_bacterium_9_1_43BFAA | No | 0.948279 | 0.004243 | 0.039171 |
| Bifidobacterium_angulatum | No | 1.465646 | 0.004353 | 0.039826 |
| Ruminococcaceae_bacterium_Marseille.P2935 | No | 0.895219 | 0.004417 | 0.04024 |
| Lachnospiraceae_bacterium_3_1_46FAA | No | 0.707702 | 0.004463 | 0.040306 |
| Provencibacterium_massiliense | No | 0.934103 | 0.004672 | 0.041307 |
| Faecalitalea_cylindroides | No | 0.575337 | 0.004686 | 0.041307 |
| Coprococcus_sp._HPP0048 | No | 1.048614 | 0.004723 | 0.041307 |
| Parabacteroides_sp._SN4 | No | 0.664894 | 0.004817 | 0.041342 |
| Adlercreutzia_equolifaciens | No | 1.151929 | 0.004847 | 0.041427 |
| Dielma_fastidiosa | No | 3.517332 | 0.004924 | 0.041565 |
| Erysipelotrichaceae_bacterium_6_1_45 | No | 0.559721 | 0.004932 | 0.041565 |
| Eubacterium_hallii | No | 0.528111 | 0.004992 | 0.041805 |
| Coprococcus_sp._HPP0074 | No | 0.830471 | 0.005025 | 0.041919 |
| Fusobacterium_naviforme | No | 1.734117 | 0.005546 | 0.044825 |
| Paraprevotella_xylaniphila | No | 0.870895 | 0.005615 | 0.045204 |
| Prevotella_sp._Marseille.P4119 | No | 0.756948 | 0.005651 | 0.04532 |
| Bacteroides_salyersiae | No | 0.790116 | 0.005748 | 0.045906 |
| Bacteroides_sp._3_1_33FAA | No | 0.676546 | 0.005811 | 0.046064 |
| Clostridium_glycyrrhizinilyticum | No | 0.607986 | 0.005986 | 0.047096 |
| Bacteroides_sp._9_1_42FAA | No | 0.603241 | 0.006032 | 0.047171 |
| Clostridium_asparagiforme | No | 0.694766 | 0.006085 | 0.047276 |
| Bifidobacterium_bifidum | No | 0.70276 | 0.006229 | 0.047741 |
| Bacteroides_stercoris | No | 0.446073 | 0.006293 | 0.048053 |
| Ruminococcus_bromii | No | 1.611246 | 0.006333 | 0.048184 |
| Traorella_massiliensis | No | 0.715533 | 0.006428 | 0.048413 |
| Lachnoanaerobaculum_sp._OBRC5.5 | No | 1.099442 | 0.006433 | 0.048413 |

Endpoint: Composite score of decompensation: 0=compensated, 1=HE only, 2=Ascites only, 3=both HE and ascites, 4=infected

**Table S7: MAAsLin2 for death and hospitalizations using VFs only from six pathogens**

| **Hospitalizations** | **Coefficient** | **p-value** | **q-value** |
| --- | --- | --- | --- |
| **MELD score** | 0.482547 | 9.58E-13 | 4.23E-10 |
| **Endpoint** | 0.68787 | 1.68E-10 | 3.71E-08 |
| **Hepatic encephalopathy** | 0.621688 | 7.67E-07 | 1.13E-04 |
| **Lactulose use** | 0.653926 | 2.78E-06 | 3.07E-04 |
| Atl (*Staphylococcus aureus autolysin*) | 3.905132 | 8.33E-04 | 0.073632 |
| AIDA_I_type_C (*Enterobacteriaceae*) | 3.975208 | 0.001459 | 0.106223 |
| BopD (*Enterococcus* *biofilm*) | 0.827387 | 0.002285 | 0.106223 |
| Cell_wall_associated_fibronectin_binding_protein (*Staphylococcus)* | 1.195622 | 0.002347 | 0.106223 |
| Fibronectin_binding_protein (*Staphylococcus)* | 3.553583 | 0.002447 | 0.106223 |
| **Death** | **Coefficient** | **p-value** | **q-value** |
| **Endpoint** | 0.682565 | 4.08E-08 | 1.80E-05 |
| **MELD score** | 0.500582 | 1.49E-07 | 3.30E-05 |
| **Lactulose use** | 0.66683 | 3.56E-05 | 0.00393 |
| Hepatic Encephalopathy | 0.595509 | 5.31E-05 | 0.004692 |
| BopD (*Enterococcus* *biofilm*) | 0.961648 | 0.001393 | 0.04224 |
| LepA (*Pseudomonas*) | 3.624885 | 0.002587 | 0.060192 |
| Ebp_pili (*Enterococcus* pili) | 0.933116 | 0.003501 | 0.073467 |
| EstA (*Streptococcus)* | 3.474077 | 0.003859 | 0.074165 |
| Streptococcal_collagen_like_proteins (*Streptococcus*) | 0.698775 | 0.007558 | 0.091203 |

Endpoint: Composite score of decompensation: 0=compensated, 1=HE only, 2=Ascites only, 3=both HE and ascites, 4=infected, Bold are clinical factors

Table S8: Baseline characteristics of subjects

|  | FMT (n=10) | Placebo (n=10) | P value |
| --- | --- | --- | --- |
| Age | 63.3±4.2 | 64.2±6.2 | 0.71 |
| Gender (M/F) | 8/2 | 8/2 | 1.0 |
| Race (Caucasian/African-American/Hispanic) | 7/3/0 | 7/3/0 | 1.0 |
| Etiology of cirrhosis (HCV/Alcohol/HCV+  Alcohol/NASH/Others) | 2/1/3/2/2 | 3/1/2/2/1 | 0.78 |
| PPI use | 10 | 10 | 1.0 |
| Lactulose | 10 | 10 | 1.0 |
| Rifaximin | 10 | 10 | 1.0 |
| MELD score | 9.5±2.6 | 10.9±4.2 | 0.39 |
| AST | 48.4±13.8 | 40.9±20.8 | 0.36 |
| ALT | 39.0±16.4 | 21.9±15.5 | 0.33 |
| Alkaline Phosphatase | 144.8±66.7 | 126.9±40.7 | 0.48 |
| INR | 1.27±0.17 | 1.30±0.16 | 0.69 |
| Bilirubin | 1.26±0.80 | 1.46±0.80 | 0.53 |
| Serum albumin | 3.3±0.5 | 3.3±0.6 | 0.67 |
| WBC (10^3^/ml) | 4.65±1.43 | 5.4±2.0 | 0.37 |
| Hemoglobin (g/dl) | 13.5±1.9 | 12.9±2.6 | 0.21 |
| Platelet count(10^3^/ml) | 113.0±48.5 | 140.4±70.5 | 0.33 |

Table S9: Changes in laboratory parameters over time between groups

|  | Safety visit | | P value | 30-day visit | | P value |
| --- | --- | --- | --- | --- | --- | --- |
|  | Placebo | FMT |  | Placebo | FMT |  |
| MELD score | 11.7±3.9 | 10.2±4.5 | 0.44 | 11.3±3.9 | 8.7±2.9 | 0.11 |
| AST | 41.0±21.4 | 49.4±11.1 | 0.29 | 43.4±26.0 | 50.2±16.3 | 0.50 |
| ALT | 30.9±12.4 | 39.0±13.2 | 0.18 | 35.1±14.6 | 40.4±13.6 | 0.42 |
| Alkaline Phosphatase | 133.7±57.3 | 145.8±67.0 | 0.67 | 132.2±57.6 | 132.9±59.7 | 0.98 |
| INR | 1.32±0.22 | 1.29±0.16 | 0.73 | 1.31±0.23 | 1.23±0.14 | 0.40 |
| Bilirubin | 1.57±0.72 | 1.49±0.74 | 0.81 | 1.62±0.92 | 1.29±0.59 | 0.336 |
| Serum albumin | 3.17±0.57 | 3.33±0.48 | 0.51 | 3.32±0.67 | 3.52±0.49 | 0.46 |
| WBC (10^3^/ml) | 5.1±2.2 | 4.9±1.5 | 0.83 | 5.1±2.0 | 5.0±1.3 | 0.93 |
| Hemoglobin (g/dl) | 11.7±2.8 | 13.4±1.8 | 0.13 | 12.5±2.9 | 13.9±1.5 | 0.20 |
| Platelet count(10^3^/ml) | 146.4±85.6 | 108.4±31.8 | 0.22 | 142.9±76.5 | 116.0±53.3 | 0.38 |

Safety visit is 1-2 weeks after the initial intervention; comparisons performed using unpaired t-tests

Table S10 bacterial Species on MAAsLin2 rifaximin versus not

| Feature | On Rifaximin? | feature | direction | coef |
| --- | --- | --- | --- | --- |
| HE not | Yes | 1.019831 | 2.48E-14 | 2.57E-11 |
| Lactulose use | Yes | 1.176699 | 1.85E-14 | 2.57E-11 |
| Endpoint | Yes | 0.684238 | 4.75E-10 | 3.29E-07 |
| Lactobacillus acidophilus | Yes | 2.122632 | 1.85E-07 | 7.66E-05 |
| Clostridium cocleatum | Yes | 2.907137 | 1.39E-06 | 4.81E-04 |
| Inediibacterium massiliense | Yes | 3.962371 | 9.48E-05 | 0.004799 |
| Bifidobacterium breve | Yes | 0.411133 | 3.52E-04 | 0.012422 |
| Bifidobacterium gallinarum | Yes | 1.048663 | 9.20E-04 | 0.020762 |
| PPI use | Yes | 0.424498 | 0.001262 | 0.026566 |
| Bifidobacterium saguini | Yes | 1.417961 | 0.001413 | 0.028772 |
| Lactobacillus rhamnosus | Yes | 1.153813 | 0.002072 | 0.036766 |
| Feature | On Rifaximin? | coefficient | P-value | Q-value |
| Clostridium_glycyrrhizinilyticum | No | 0.765574 | 1.66E-07 | 7.66E-05 |
| Adlercreutzia_equolifaciens | No | 1.283171 | 2.96E-06 | 6.82E-04 |
| Coprococcus_eutactus | No | 0.653805 | 2.90E-06 | 6.82E-04 |
| Eubacterium_ventriosum | No | 0.678073 | 2.80E-06 | 6.82E-04 |
| Clostridium_sp._AT4 | No | 0.737925 | 4.05E-06 | 7.72E-04 |
| Lachnospiraceae_bacterium_1_4_56FAA | No | 0.743979 | 4.09E-06 | 7.72E-04 |
| Blautia_hydrogenotrophica | No | 0.948044 | 6.53E-06 | 0.001036 |
| Blautia_obeum | No | 0.462221 | 6.37E-06 | 0.001036 |
| Blautia_sp._SF.50 | No | 0.598614 | 6.98E-06 | 0.001036 |
| Lachnospira_pectinoschiza | No | 0.726109 | 8.68E-06 | 0.001158 |
| Roseburia_hominis | No | 0.631609 | 8.92E-06 | 0.001158 |
| Blautia_sp._KLE_1732 | No | 0.620797 | 1.15E-05 | 0.001407 |
| Alistipes_finegoldii | No | 0.596891 | 1.23E-05 | 0.001413 |
| Anaerostipes_hadrus | No | 0.557002 | 1.70E-05 | 0.001765 |
| Eubacterium_hallii | No | 0.527731 | 1.68E-05 | 0.001765 |
| Gordonibacter_pamelaeae | No | 0.991401 | 1.92E-05 | 0.001893 |
| Roseburia_intestinalis | No | 0.503429 | 2.50E-05 | 0.002361 |
| Eggerthella_lenta | No | 0.586761 | 2.71E-05 | 0.002446 |
| Faecalicatena_contorta | No | 0.663681 | 3.02E-05 | 0.00251 |
| Tyzzerella_nexilis | No | 0.515321 | 2.99E-05 | 0.00251 |
| Blautia_massiliensis | No | 0.398415 | 3.58E-05 | 0.002654 |
| Blautia_sp._Marseille.P2398 | No | 0.519262 | 3.55E-05 | 0.002654 |
| Blautia_sp._Marseille.P3087 | No | 0.608349 | 3.71E-05 | 0.002654 |
| Roseburia_inulinivorans | No | 0.475086 | 3.39E-05 | 0.002654 |
| Bacteroides_pectinophilus | No | 0.58466 | 4.10E-05 | 0.002697 |
| Blautia_wexlerae | No | 0.416219 | 4.01E-05 | 0.002697 |
| Mordavella_sp._Marseille.P3756 | No | 0.601957 | 4.16E-05 | 0.002697 |
| Anaeromassilibacillus_sp._Marseille.P3371 | No | 0.605539 | 4.30E-05 | 0.002705 |
| Alistipes_putredinis | No | 0.572988 | 4.49E-05 | 0.00274 |
| Eubacterium_eligens | No | 0.529023 | 5.16E-05 | 0.00306 |
| Clostridium_sp._SS2.1 | No | 0.782288 | 6.81E-05 | 0.003927 |
| Ruminococcaceae_bacterium_cv2 | No | 0.603224 | 7.86E-05 | 0.004409 |
| Ruthenibacterium_lactatiformans | No | 0.494858 | 8.32E-05 | 0.004546 |
| Dorea_longicatena | No | 0.427285 | 8.65E-05 | 0.004604 |
| Alistipes_onderdonkii | No | 0.69832 | 9.33E-05 | 0.004799 |
| Blautia_producta | No | 0.670889 | 1.03E-04 | 0.00499 |
| Eubacterium_ramulus | No | 0.440789 | 1.03E-04 | 0.00499 |
| Eubacterium_rectale | No | 0.410984 | 1.09E-04 | 0.005128 |
| Clostridium_sp._M62.1 | No | 0.470602 | 1.13E-04 | 0.005225 |
| Negativibacillus_massiliensis | No | 0.686117 | 1.25E-04 | 0.005639 |
| Roseburia_faecis | No | 0.531993 | 1.28E-04 | 0.005639 |
| Clostridium_disporicum | No | 2.539114 | 1.33E-04 | 0.005668 |
| Dorea_formicigenerans | No | 0.428456 | 1.34E-04 | 0.005668 |
| Johnsonella_ignava | No | 0.780777 | 1.42E-04 | 0.005907 |
| Blautia_sp._Marseille.P3201T | No | 0.56087 | 1.51E-04 | 0.006062 |
| Subdoligranulum_sp._4_3_54A2FAA | No | 0.473376 | 1.52E-04 | 0.006062 |
| Anaerotruncus_colihominis | No | 0.427988 | 1.57E-04 | 0.006142 |
| Butyrivibrio_crossotus | No | 0.618432 | 1.75E-04 | 0.00674 |
| Ruminococcus_faecis | No | 0.510616 | 2.13E-04 | 0.008043 |
| Eubacterium_sp._SB2 | No | 1.206943 | 2.25E-04 | 0.008328 |
| Alistipes_ihumii | No | 1.096431 | 2.54E-04 | 0.009239 |
| Alistipes_sp._CHKCI003 | No | 1.017553 | 3.60E-04 | 0.012422 |
| Lactobacillus_rogosae | No | 0.699313 | 3.69E-04 | 0.012422 |
| Prevotella_disiens | No | 1.651812 | 3.58E-04 | 0.012422 |
| Subdoligranulum_variabile | No | 0.446166 | 3.71E-04 | 0.012422 |
| Lachnospiraceae_bacterium_5_1_63FAA | No | 0.611298 | 3.84E-04 | 0.012651 |
| Clostridium_sp._HMSC19A11 | No | 1.493922 | 3.99E-04 | 0.012932 |
| Ruminococcus_callidus | No | 0.870348 | 4.18E-04 | 0.013359 |
| Alistipes_shahii | No | 0.541964 | 4.72E-04 | 0.014411 |
| Gordonibacter_urolithinfaciens | No | 1.155134 | 4.68E-04 | 0.014411 |
| Ruminococcus_torques | No | 0.367832 | 4.61E-04 | 0.014411 |
| Actinomyces_naeslundii | No | 1.475869 | 5.24E-04 | 0.0148 |
| Alistipes_sp._AL.1 | No | 0.769331 | 5.03E-04 | 0.0148 |
| Clostridium_scindens | No | 0.702706 | 5.42E-04 | 0.0148 |
| Dialister_invisus | No | 0.655419 | 5.18E-04 | 0.0148 |
| Fusicatenibacter_saccharivorans | No | 0.35455 | 5.42E-04 | 0.0148 |
| Holdemania_filiformis | No | 0.533406 | 4.97E-04 | 0.0148 |
| Prevotella_buccalis | No | 1.146145 | 5.31E-04 | 0.0148 |
| Romboutsia_timonensis | No | 3.47238 | 5.12E-04 | 0.0148 |
| Collinsella_bouchesdurhonensis | No | 1.148072 | 5.57E-04 | 0.015008 |
| Collinsella_sp._4_8_47FAA | No | 0.547951 | 5.89E-04 | 0.015611 |
| Lachnospiraceae_bacterium_3_1_46FAA | No | 0.538553 | 5.94E-04 | 0.015611 |
| Phocea_massiliensis | No | 0.544125 | 6.28E-04 | 0.016295 |
| Fusicatenibacter_sp._2789STDY5834925 | No | 0.711523 | 6.38E-04 | 0.016339 |
| Clostridium_leptum | No | 0.512713 | 6.56E-04 | 0.016406 |
| Lachnospiraceae_bacterium_9_1_43BFAA | No | 0.654266 | 6.55E-04 | 0.016406 |
| Clostridioides_difficile | No | 0.49435 | 7.49E-04 | 0.018298 |
| Eggerthella_sp._1_3_56FAA | No | 0.556636 | 7.52E-04 | 0.018298 |
| Sellimonas_intestinalis | No | 0.706904 | 0.000758 | 0.018298 |
| Actinomyces_oris | No | 0.783704 | 7.91E-04 | 0.018663 |
| Eisenbergiella_massiliensis | No | 0.45861 | 7.87E-04 | 0.018663 |
| Lachnospiraceae_bacterium_3_1_57FAA_CT1 | No | 0.657293 | 8.37E-04 | 0.019519 |
| Lachnospiraceae_bacterium_COE1 | No | 0.882302 | 8.79E-04 | 0.020277 |
| Blautia_hansenii | No | 0.479205 | 8.93E-04 | 0.020361 |
| Clostridium_sp._ATCC_29733 | No | 0.496529 | 9.86E-04 | 0.022015 |
| Emergencia_timonensis | No | 0.608694 | 0.001112 | 0.024561 |
| Bacteroides_sp._Marseille.P3208T | No | 0.733575 | 0.001134 | 0.02478 |
| Collinsella_aerofaciens | No | 0.409311 | 0.00119 | 0.025744 |
| Bacteroides_sp._AR29 | No | 0.495883 | 0.001267 | 0.026566 |
| Bacteroides_sp._D20 | No | 0.476161 | 0.001255 | 0.026566 |
| Lachnospiraceae_bacterium_A2 | No | 0.595704 | 0.001281 | 0.026598 |
| Enterorhabdus_mucosicola | No | 1.753236 | 0.001428 | 0.028772 |
| Lachnospiraceae_bacterium_2_1_46FAA | No | 0.793418 | 0.001415 | 0.028772 |
| Bacteroides_finegoldii | No | 0.362196 | 0.001443 | 0.02881 |
| Bacteroides_bouchesdurhonensis | No | 0.468662 | 0.001482 | 0.029307 |
| Clostridiales_bacterium_KLE1615 | No | 0.485893 | 0.001529 | 0.029948 |
| Clostridium_bolteae | No | 0.381402 | 0.001563 | 0.03033 |
| Acetivibrio_ethanolgignens | No | 0.559707 | 0.001588 | 0.030516 |
| Clostridium_saccharolyticum | No | 0.415757 | 0.001685 | 0.03152 |
| Hespellia_stercorisuis | No | 0.642038 | 0.001659 | 0.03152 |
| Lachnospiraceae_bacterium_3.1 | No | 0.800332 | 0.001685 | 0.03152 |
| Streptococcus_agalactiae | No | 0.865353 | 0.001781 | 0.03302 |
| Alistipes_senegalensis | No | 0.59606 | 0.001825 | 0.033523 |
| Agathobaculum_desmolans | No | 0.420972 | 0.001961 | 0.03558 |
| Prevotella_sp._Marseille.P4119 | No | 0.535284 | 0.001971 | 0.03558 |
| Clostridium_symbiosum | No | 0.343818 | 0.00199 | 0.035607 |
| Coprococcus_sp._HPP0048 | No | 0.65243 | 0.002118 | 0.036908 |
| Enterorhabdus_caecimuris | No | 2.240336 | 0.002105 | 0.036908 |
| Monoglobus_pectinilyticus | No | 0.763433 | 0.002133 | 0.036908 |
| Bacteroides_sp._4_1_36 | No | 0.478748 | 0.002161 | 0.037077 |
| Streptococcus_sp._C150 | No | 0.68266 | 0.002231 | 0.037513 |
| Tidjanibacter_massiliensis | No | 1.071115 | 0.002241 | 0.037513 |
| Traorella_massiliensis | No | 0.485602 | 0.00223 | 0.037513 |
| Odoribacter_splanchnicus | No | 0.492865 | 0.00238 | 0.039211 |
| Ruminococcus_bromii | No | 0.842198 | 0.002372 | 0.039211 |
| Holdemania_sp._Marseille.P2844 | No | 0.683501 | 0.002531 | 0.041379 |
| Ileibacterium_massiliense | No | 0.768647 | 0.002575 | 0.041756 |
| Christensenella_massiliensis | No | 0.829663 | 0.002635 | 0.04241 |
| Lactobacillus_aviarius | No | 0.958945 | 0.002827 | 0.04514 |
| Catabacter_hongkongensis | No | 1.345575 | 0.002995 | 0.047464 |
| Coprococcus_sp._HPP0074 | No | 0.519761 | 0.003056 | 0.048055 |
| Oribacterium_sp._oral_taxon_078 | No | 0.907217 | 0.003158 | 0.049287 |
| Actinomyces_dentalis | No | 1.64429 | 0.003182 | 0.049297 |

Endpoint: Composite score of decompensation: 0=compensated, 1=HE only, 2=Ascites only, 3=both HE and ascites, 4=infected. HE: hepatic encephalopathy

Figure S11 Virulence factor changes in those on or not on rifaximin using MAAslin2

| Feature | On Rifaximin? | Coefficient | P-value | Q-Value |
| --- | --- | --- | --- | --- |
| Catalase..CVF760. | No | 0.534224 | 2.84E-06 | 8.83E-04 |
| Colibactin..VF0573. | No | 0.875921 | 2.30E-05 | 0.005724 |
| HE not | Yes | 1.041454 | 2.05E-14 | 1.43E-11 |
| Lactulose use | Yes | 1.179973 | 2.31E-14 | 1.43E-11 |
| Endpoint | Yes | 0.715853 | 1.90E-10 | 7.86E-08 |

Endpoint: Composite score of decompensation: 0=compensated, 1=HE only, 2=Ascites only, 3=both HE and ascites, 4=infected. HE: hepatic encephalopathy
