# Supplementary material for: Multiple bacterial virulence factors focused on adherence and biofilm formation associate with outcomes in cirrhosis
Source: Gut Microbes. 2021 Nov 8;13(1):1993584. doi: 10.1080/19490976.2021.1993584 (PMC8582993; doi:10.1080/19490976.2021.1993584)
Supplement: Supplemental Material [file KGMI_A_1993584_SM6599.zip › VF Supplementary figures for GUT MICROBES 9_26_21.pptx]

## Slide 1
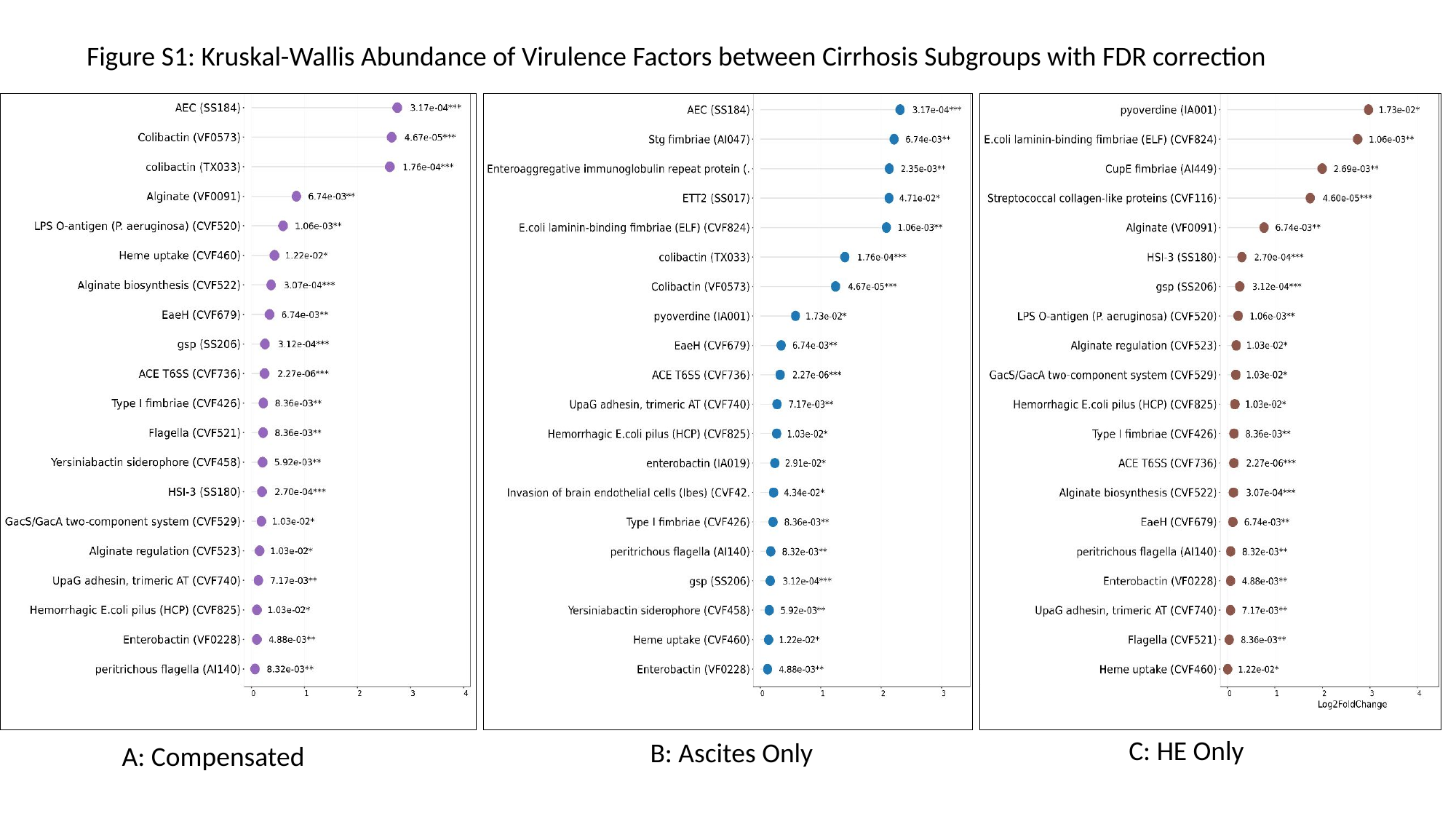

Figure S1: Kruskal-Wallis Abundance of Virulence Factors between Cirrhosis Subgroups with FDR correction
C: HE Only
B: Ascites Only
A: Compensated

## Slide 2
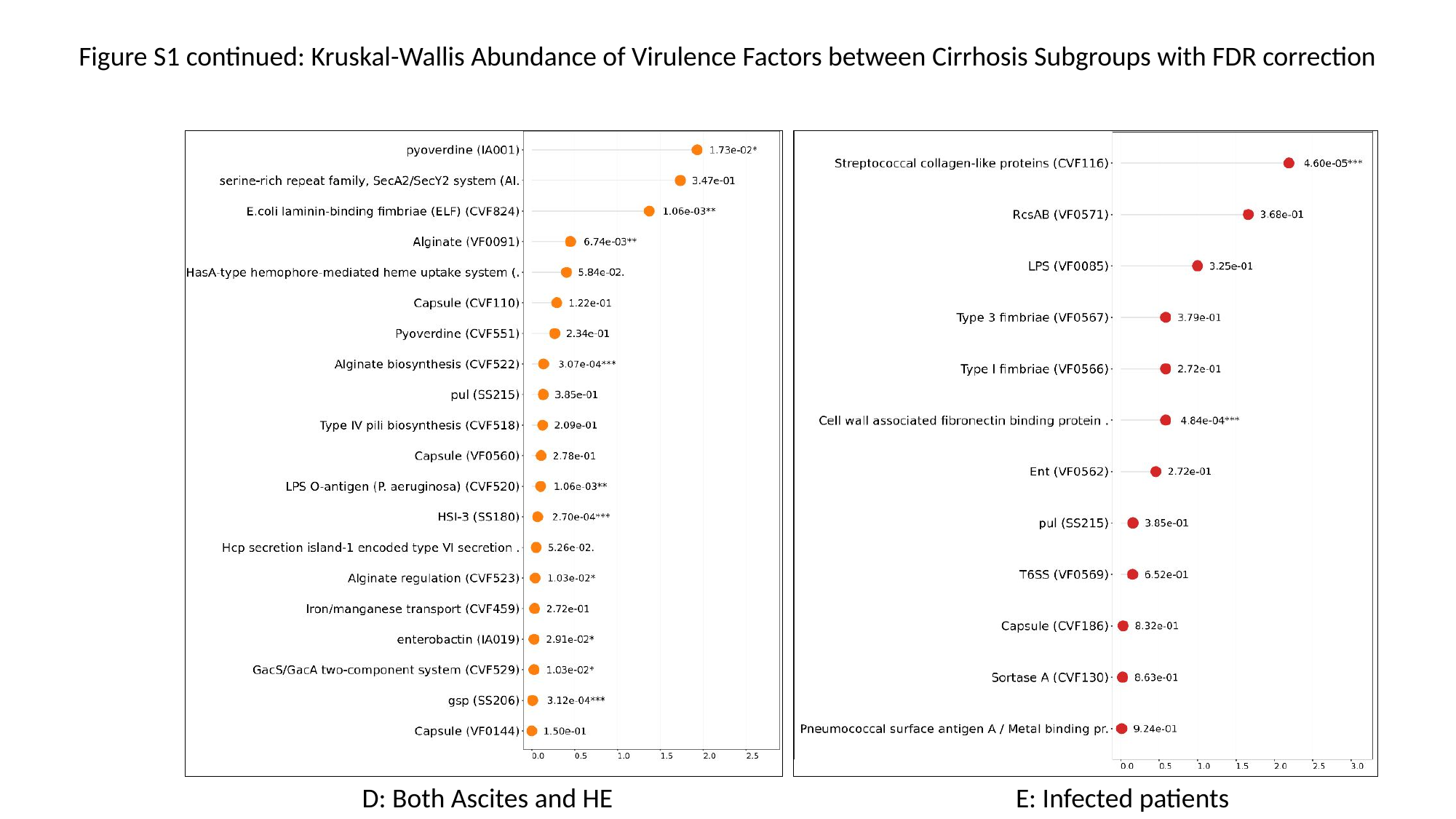

Figure S1 continued: Kruskal-Wallis Abundance of Virulence Factors between Cirrhosis Subgroups with FDR correction
D: Both Ascites and HE
E: Infected patients

## Slide 3
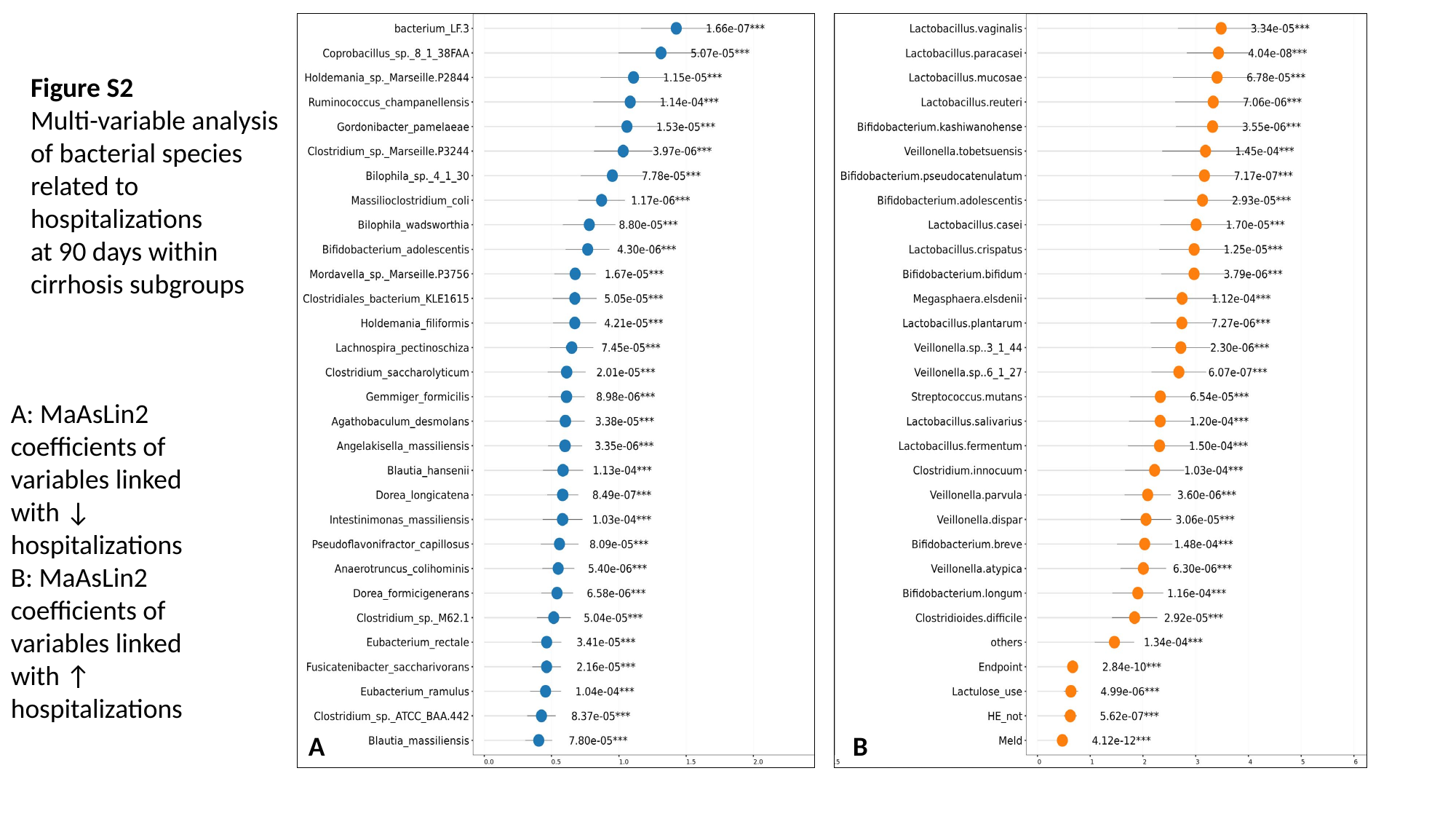

Figure S2
Multi-variable analysis
of bacterial species
related to
hospitalizations
at 90 days within
cirrhosis subgroups
A: MaAsLin2 coefficients of variables linked with ↓ hospitalizations
B: MaAsLin2 coefficients of variables linked with ↑ hospitalizations
A
B

## Slide 4
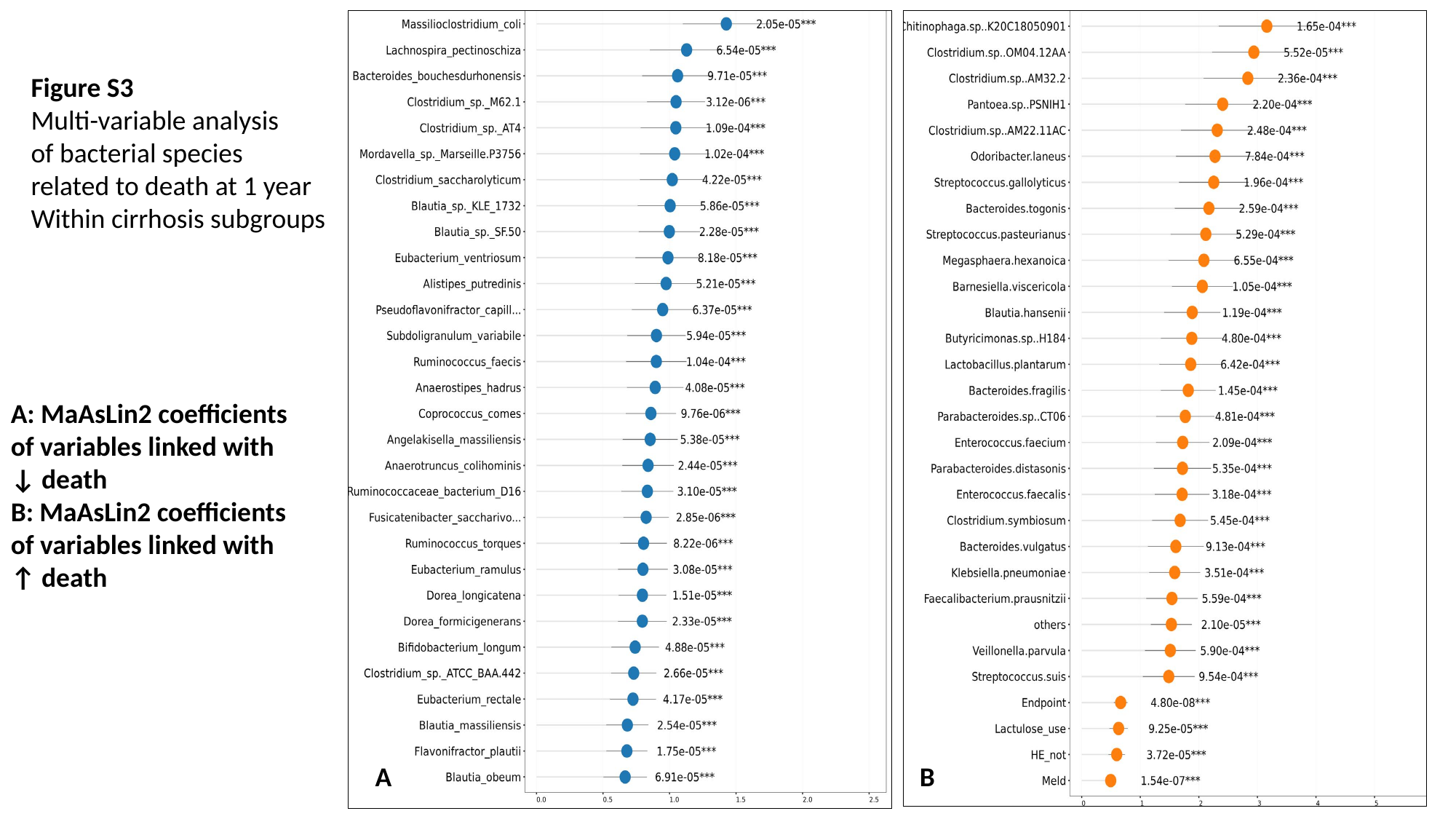

Figure S3
Multi-variable analysis
of bacterial species
related to death at 1 year
Within cirrhosis subgroups
A: MaAsLin2 coefficients of variables linked with ↓ death
B: MaAsLin2 coefficients of variables linked with ↑ death
A
B

## Slide 5
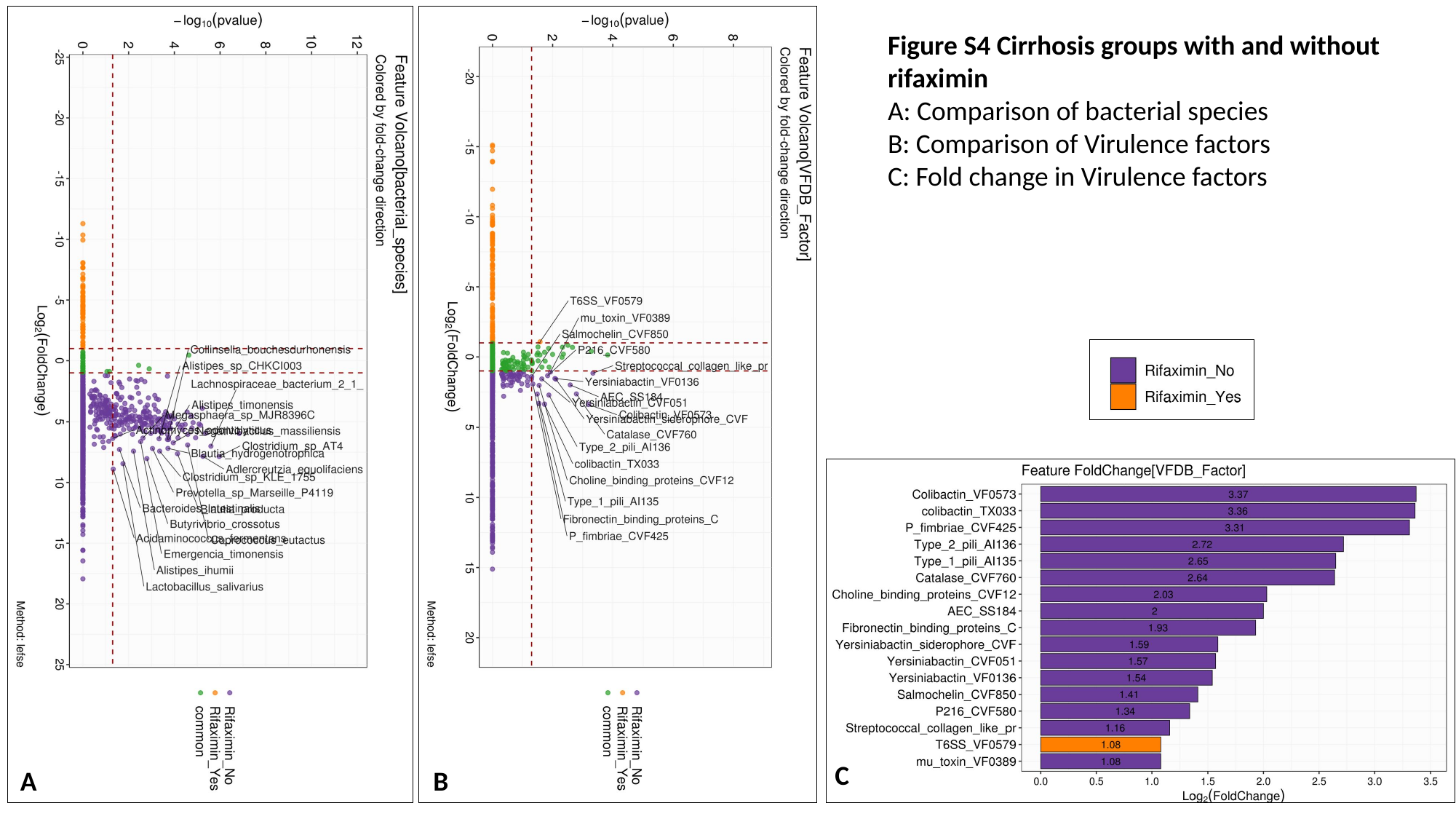

Figure S4 Cirrhosis groups with and without
rifaximin
A: Comparison of bacterial species
B: Comparison of Virulence factors
C: Fold change in Virulence factors
C
A
B
